# Structural architecture and brain network efficiency link polygenic scores to intelligence

**DOI:** 10.1002/hbm.26286

**Published:** 2023-04-04

**Authors:** Erhan Genç, Dorothea Metzen, Christoph Fraenz, Caroline Schlüter, Manuel C. Voelkle, Larissa Arning, Fabian Streit, Huu Phuc Nguyen, Onur Güntürkün, Sebastian Ocklenburg, Robert Kumsta

**Affiliations:** ^1^ Department of Psychology and Neuroscience Leibniz Research Centre for Working Environment and Human Factors (IfADo) Dortmund Germany; ^2^ Biopsychology, Institute for Cognitive Neuroscience, Faculty of Psychology Ruhr University Bochum Bochum Germany; ^3^ Psychological Research Methods Department of Psychology Humboldt University Berlin Germany; ^4^ Department of Human Genetics, Faculty of Medicine Ruhr University Bochum Bochum Germany; ^5^ Department Genetic Epidemiology in Psychiatry, Central Institute of Mental Health, Medical Faculty Mannheim University of Heidelberg Mannheim Germany; ^6^ Department of Psychology Medical School Hamburg Hamburg Germany; ^7^ ICAN Institute for Cognitive and Affective Neuroscience Medical School Hamburg Hamburg Germany; ^8^ Genetic Psychology, Faculty of Psychology Ruhr University Bochum Bochum Germany; ^9^ Department of Behavioural and Cognitive Sciences, Laboratory for Stress and Gene‐Environment Interplay University of Luxembourg Esch‐sur‐Alzette Luxembourg

**Keywords:** DWI connectivity, graph theory, intelligence, P‐FIT, polygenic scores, resting state fMRI

## Abstract

Intelligence is highly heritable. Genome‐wide association studies (GWAS) have shown that thousands of alleles contribute to variation in intelligence with small effect sizes. Polygenic scores (PGS), which combine these effects into one genetic summary measure, are increasingly used to investigate polygenic effects in independent samples. Whereas PGS explain a considerable amount of variance in intelligence, it is largely unknown how brain structure and function mediate this relationship. Here, we show that individuals with higher PGS for educational attainment and intelligence had higher scores on cognitive tests, larger surface area, and more efficient fiber connectivity derived by graph theory. Fiber network efficiency as well as the surface of brain areas partly located in parieto‐frontal regions were found to mediate the relationship between PGS and cognitive performance. These findings are a crucial step forward in decoding the neurogenetic underpinnings of intelligence, as they identify specific regional networks that link polygenic predisposition to intelligence.

## Graphical abstract

We investigated the mediating effect of surface area, cortical thickness as well as fiber and functional network efficiency on the effect of polygenic scores on intelligence. Fiber network efficiency as well as the surface of brain areas partly located in parieto‐frontal regions were found to mediate the relationship between PGS and cognitive performance.

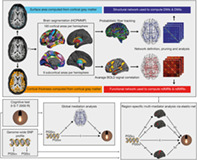

## 1 INTRODUCTION

Intelligence is a general mental capability that involves the ability to reason, plan, solve problems, and learn from experience (Deary et al., 2021). General intelligence, or *g*, is one of the most intensely studied psychological phenotypes for its high stability across the life course (Deary, 2014) and its high predictive value for educational success (Deary et al., 2007) and health outcomes (Calvin et al., 2017). Despite intelligence's high relevance in everyday life, investigating its neurogenetic underpinnings showed to be surprisingly challenging (Plomin & von Stumm, 2018).

Intelligence is a highly heritable trait (Plomin & von Stumm, 2018), with about 50% of the variance accounted for by genetic factors. Genome‐wide association studies (GWAS), which test the association between single nucleotide polymorphisms (SNPs) and a phenotype, showed that intelligence is highly polygenic, with thousands of alleles across the genome contributing with small effect sizes (Savage et al., 2018). One way forward in accounting for this highly polygenic architecture is to combine the effects of different SNPs across the whole genome into one summary measure, so‐called polygenic scores (PGS) (Choi et al., 2020). PGS are determined by computing the sum of allelic effects for a specific phenotype such as intelligence over the whole genome and weighting them with an effect size estimate obtained from GWAS. Importantly, PGS use the statistical power of well‐powered GWAS of discovery samples to be applied robustly in smaller target samples (Dima & Breen, 2015; Dudbridge, 2013). In the case of intelligence, PGS derived from one of the largest GWAS to date (Savage et al., 2018) explain up to 5.2% of variance in general intelligence. For educational attainment—highly correlated to intelligence and more readily available—larger GWAS could be realized, with resulting PGS that explain up to 11% of the variance in educational attainment (Lee et al., 2018), and 7% of variance in intelligence (Plomin & von Stumm, 2018).

In addition, PGS can be leveraged to map the pathway from genetic disposition to phenotype. Whereas it is known that intelligence is influenced by brain structure and function as well as network efficiency (Barbey, 2018; Deary et al., 2010), a functional understanding of which specific brain parameters mediate the link between genetic variation and intelligence is missing. Several brain properties are related to intelligence, including brain volume, surface area, and cortical thickness (Choi et al., 2008; McDaniel, 2005; Narr et al., 2007; Pietschnig et al., 2015). Importantly, intelligence is not tied to the properties of one single brain area, but to a wide network of brain areas spread across the whole cortex. Here, a network mainly comprising the dorsolateral prefrontal cortex, the parietal lobe, the anterior cingulate cortex, the temporal lobe, and the occipital lobe seems to be central for cognitive performance, as proposed by the Parieto‐Frontal Integration Theory of intelligence (P‐FIT) (Jung & Haier, 2007). The theory assumes that all of these P‐FIT areas, even though they were identified independently of each other, are likely to have strong interconnections and form an extensive brain network. Recent studies and models focusing on connectivity‐based approaches indicate that there may be brain areas whose structural and functional properties are not related to intelligence, while their connectivity patterns are (Barbey, 2018; Fraenz et al., 2021). Previous research, in which the connectivity between brain regions was quantified via diffusion‐weighted imaging (DWI) and graph theoretical approaches, showed that the brain's global efficiency as well as the nodal efficiency of brain areas from the P‐FIT network and beyond are associated with intelligence (Fischer et al., 2014; Kim et al., 2016; Li et al., 2009; Ma et al., 2017; Pineda‐Pardo et al., 2016; Wen et al., 2011; Wiseman et al., 2018). The largest study investigating associations of intelligence and structural brain properties found associations of *β* = .276 for total brain volume and of *β* = .0246 for white matter volume (Cox et al., 2019). On a regional level, associations with cortical volume of frontal areas were largest.

In addition to structural connectivity, graph theory can also be used in combination with data from resting‐state fMRI in order to study the brain's functional connectivity (Fox & Raichle, 2007). There is evidence that general intelligence is positively correlated with functional global efficiency (van den Heuvel et al., 2009) and the nodal efficiency of areas belonging to the P‐FIT network. However, subsequent studies could not replicate these associations (Hilger et al., 2017a, 2017b; Kruschwitz et al., 2018). Thus, structural properties of the P‐FIT network seem to show a more reliable correlation to intelligence than functional properties.

Macrostructural properties of specific brain areas and the structural efficiency of the human connectome represent likely candidates for mediating the effects of genetic variation on general intelligence. Several GWAS reporting genetic correlations between brain properties and intelligence, that is, overlapping genetic variants being associated with both phenotypes, support this notion (Cheng et al., 2020; Feng et al., 2020; Ge et al., 2019; Grasby et al., 2020; Lee et al., 2019; Zhao, Li, et al., 2021; Zhao, Zhang, et al., 2021). In a complementary approach, studies demonstrated associations between PGS for educational attainment or general intelligence and brain properties (Jansen et al., 2019; Knol et al., 2019; Loughnan et al., 2021). However, mediation analyses that measure polygenic disposition, brain properties (putative mediator) and intelligence (outcome) in the same sample are rare. By doing so, one can directly analyze the extent to which the association between PGS and intelligence is explained via variation in brain structure and function. Three studies to date have investigated the mediation effect on the macrostructural level (Elliott et al., 2019; Lett et al., 2020; Mitchell et al., 2020). Elliott et al. (2019) analyzed the potential mediation effect of total brain volume on the relationship between PGS for educational attainment and cognitive performance. They found that participants with larger brains and with higher PGS performed better on cognitive tests. PGS were also positively associated with brain size. However, there was no clear overall mediation effect of brain volume. Since general intelligence is associated with specific regions in the brain, subsequent studies focused on region‐specific mediation effects of cortical thickness and surface area. Lett et al. (2020) employed PGS for general intelligence and found that the association between PGS and general intelligence was partially mediated by surface area and cortical thickness in prefrontal regions, anterior cingulate, insula, and medial temporal cortex. It is noteworthy that some of these regions are part of the P‐FIT network. Results were consistent across two independent samples, indicating that macrostructural properties of specific areas, partly belonging to the P‐FIT network, may indeed play a crucial role with regard to the link between genetic variation and general intelligence. Another study by Mitchell et al. (2020), which employed PGS for educational attainment, reported similar findings. They observed that surface area and cortical thickness of specific cortical regions partially mediated the effects of PGS on cognitive test performance. These regions were the fusiform gyrus, entorhinal cortex, banks of the superior temporal sulcus, the inferior frontal gyrus, and the medial orbital frontal gyrus.

To summarize, there is evidence that specific gray matter macrostructural properties of brain areas from the P‐FIT network represent likely candidates to explain the link between genetic variation and intelligence. What is missing, however, is a systems view taking into account white matter connectivity as well as functional network properties. Our study aimed to fill this crucial gap in the literature by using a multilevel deep phenotyping approach, including an integrated analysis of behavioral and neuroimaging phenotypes. We investigated the effects of two different PGS on general intelligence: PGS for educational attainment (Lee et al., 2018) and PGS for general intelligence (Savage et al., 2018). We tested the mediating role of surface area, cortical thickness, white matter fiber network efficiency, and functional network efficiency on the level of the whole brain as well as for specific brain areas. Thus, this study presents the first multimodal mediation analysis that gives brain region‐specific insight into the putative links between genetics and general intelligence.

## 2 METHODS

### 2.1 Participants

Since this is the first study investigating the mediation effect of network connectivity on the relationship between PGS and intelligence, we used effect sizes from previous studies investigating the correlation between network connectivity and intelligence (Genç et al., 2019). Thus, an a‐priori test was performed using G*Power to estimate the needed number of participants. The analysis was based on a linear multiple regression analysis with a small effect size (*f* ^2^ = .04, *α* = .05, two‐tailed, power = 0.95, number of predictors = 6). The analysis computed a total sample size of 528.

Our sample consisted of 557 adults, who reported to be free from past or present neurological and/or psychological conditions. The mean age was 27.33 years (SD = 9.43; range = 18–75), we tested 283 men (mean age = 27.1, SD = 9.86) and 274 women (mean age = 26.94, SD = 8.96). Participants were mostly university students (mean years of education = 17.4, SD = 3.12), who participated in exchange for course credit or financial compensation. The study was approved by the local ethics committee of the Faculty of Psychology at Ruhr‐University Bochum (Nr. 165). All participants gave written informed consent and were treated according to the Declaration of Helsinki. The final dataset (see 2.3) included 523 participants aged from 18 to 75 (M = 27.1, SD = 9.08, 266 women). The data is part of a large‐sample study on the neural correlates of intelligence, personality, and motivation. Hence, it has been used in other publications (Genç et al., 2018; Genç et al., 2019; Genç et al., 2021).

### 2.2 General intelligence testing: I‐S‐T 2000 R

Since participants were native German speakers, general intelligence was assessed using the basic module of the “Intelligenz‐Struktur‐Test 2000 R" (I‐S‐T 2000 R), a well‐established German intelligence test battery (Beauducel et al., 2001; Liepmann et al., 2007). The test was conducted in a quiet and well‐lit room. The I‐S‐T 2000 R comprises various types of mental test items to measure multiple facets of general intelligence and is largely comparable to the internationally established Wechsler Adult Intelligence Scale (WAIS IV) (Erdodi et al., 2017). The basic module contains 180 items assessing three sub‐factettes of general intelligence, namely verbal, numeric, and figural reasoning (Genç et al., 2021). Verbal, numeric, and figural scores are measured by three reasoning tasks with 20 items each. Examples for task‐assessing verbal abilities are sentence completion, analogies or commonalities. Numerical abilities are assessed by, for example, items on arithmetic problems and digit span tasks. Figural abilities are assessed by, for example, tasks where participants have to assemble or rotate figures mentally, match dice and solve matrix reasoning tasks. The assessment lasts about 90 minutes. The current norming sample comprises 5800 participants for the basic module (age 15–60, both sexes are represented equally). The reliability (Cronbach's *α*) of general mental ability is *α* = .96. For every participant a sum score across all 180 items was computed and used as outcome in the mediation analysis.

### 2.3 Genotyping and polygenic scores (PGS)

Exfoliated cells brushed from the oral mucosa were used for genotyping. DNA isolation was conducted with QIAamp DNA mini Kit (Qiagen GmbH, Hilden, Germany). Genotyping was performed with the Illumina Infinium Global Screening Array 1.0 with MDD and Psych content (Illumina, San Diego, CA, USA) at the Life & Brain facilities (Bonn, Germany). Filtering was done with PLINK 1.9 by eliminating all SNPs with a minor allele frequency of <0.01, missing data >0.02, or deviating from Hardy–Weinberg equilibrium by a *p*‐value <1 × 10^−6^. Subjects were excluded due to sex mismatch, > 0.02 missingness, and heterozygosity rate >|0.2|. A high quality (HWE *p* > .02, MAF >.02, missingness = 0) and LD pruned (*r* ^2^ = .01) SNP set was used for assessing relatedness and population structure. Pi hat >.2 was used to exclude subjects randomly in pairs of related subjects. Finally, we computed principal components to control for population stratification. Individuals who deviated more than 6 SD from the first 20 PCs were categorized as outliers and excluded. The final data set consisted of 523 participants and 492,348 SNPs.

We calculated genome‐wide PGS for all participants using two publicly available summary statistics: general intelligence (GI, *N* = 269,867) (Savage et al., 2018) and educational attainment (EA, *N* = 766,345) (Lee et al., 2018). PGS were calculated as weighted sums of a subject's trait‐associated alleles across all SNPs using PRSice 2.1.6. We report the best‐fit PGS, meaning that the *p*‐value threshold for PGS calculation was chosen empirically (in steps of 5*10^−5^ from 5*10^−8^ to 0.5 and for all available SNPs) so that the calculated PGS explained the maximum amount of I‐S‐T 2000 R variance (Genç et al., 2021). The best‐fit threshold selected for PGS_EA_ was 1, for PGS_GI_ it was 0.0062. The statistic “incremental *R* ^2^” was taken as a value for the predictive power of the PGS. Incremental *R* ^2^ stands for the increase in determination coefficient *R* ^2^ when the corresponding PGS is added to a regression model predicting I‐S‐T 2000 R together with our control variables. The control variables chosen were age, sex, and the first four principal components of population stratification. We used linear parametric methods for all statistical analysis in PRSice. Testing was two‐tailed (α‐level of *p* < .05). PGS_EA_ explained 3.3% of variance in I‐S‐T 2000 R score, PGS_GI_ explained 4.8%. Distributions of PGS_EA_ and PGS_GI_ are depicted in Figure S1.

### 2.4 Neuroimaging

#### 2.4.1 Acquisition of anatomical data

Magnetic resonance imaging was performed on a 3 T Philips Achieva scanner with a 32‐channel head coil. The scanner was located at Bergmannsheil University Hospital in Bochum, Germany. T1‐weighted data were obtained by means of a high‐resolution anatomical imaging sequence with the following parameters: MP‐RAGE; TR = 8.179 ms; TE = 3.7 ms; flip angle = 8°; 220 slices; matrix size = 240 × 240; resolution = 1 mm × 1 mm × 1 mm; acquisition time = 6 min.

#### 2.4.2 Acquisition of diffusion‐weighted data

Diffusion‐weighted images (DWI) were acquired using echo planar imaging with the following parameters: TR = 7652 ms, TE = 87 ms, flip angle = 90°, 60 slices, matrix size = 112 × 112, resolution = 2 mm × 2 mm × 2 mm. Diffusion weighting was carried out along 60 isotropically distributed directions with a b‐value of 1000 s/mm^2^. In addition, six volumes with a b‐value of 0 s/mm^2^ and no diffusion weighting were acquired. These served as an anatomical reference for motion correction. In total, we acquired three consecutive scans, which were averaged following the established protocol (Genç et al., 2019). This was done to increase the signal‐to‐noise ratio. Acquisition time was 30 minutes.

#### 2.4.3 Acquisition of resting‐state data

Functional MRI resting‐state images (rsfMRI) were acquired using echo planar imaging (TR = 2000 ms, TE = 30 ms, flip angle = 90°, 37 slices, matrix size = 80 × 80, resolution = 3 mm × 3 mm × 3 mm). Participants were instructed to lay still with their eyes closed and to think of nothing in particular. Acquisition time was 7 min.

### 2.5 Analysis of imaging data

#### 2.5.1 Analysis of anatomical data

**FIGURE 1:**
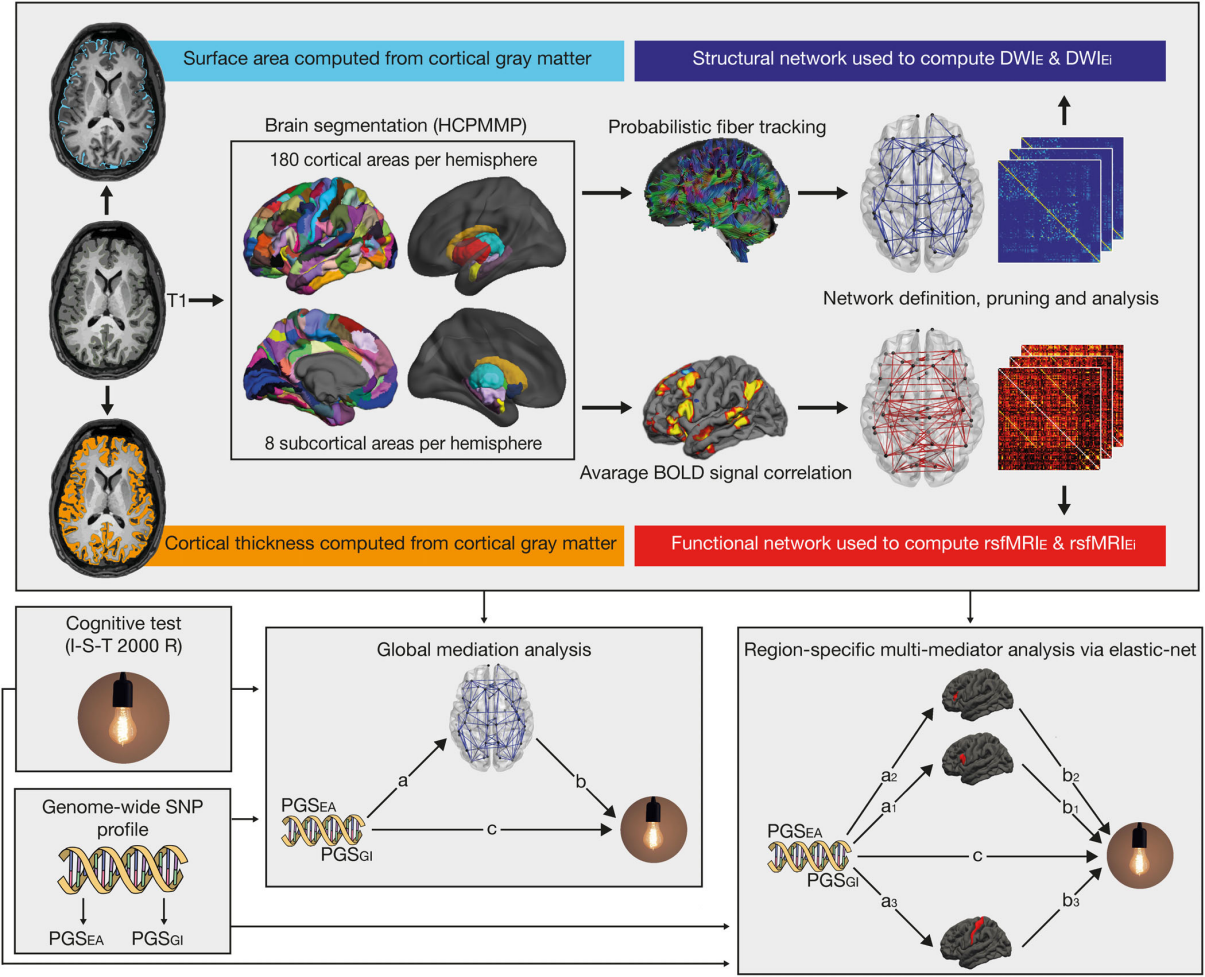
Processing steps of neurocognitive data and statistical analysis. First, T1‐weighted anatomical images were used to compute estimates of cortical surface area and cortical thickness. Second, T1‐weighted anatomical images were segmented into 180 cortical structures per hemisphere according to the HCPMMP atlas and 8 subcortical structures per hemisphere. Third, the resulting masks were linearly transformed into the native spaces of the resting‐state and diffusion‐weighted images. For the diffusion‐weighted images, probabilistic fiber tracking was carried out with the aforementioned masks serving as seed and target regions. For the resting‐state images, correlations between average BOLD time courses of all brain regions were computed. Fourth, structural and functional networks were constructed. Edges were weighted by the results of probabilistic fiber tractography or BOLD signal correlation. Fifth, these networks were used for the computation of global efficiency measures rsfMRI_E_ and DWI_E_ as well as nodal efficiency measures rsfMRI_Ei_ and DWI_Ei_. Sixth, global mediation analyses were performed for each combination of brain metric and PGS. Here, general intelligence as quantified by the I‐S‐T 2000 R sum score served as the dependent variable. Independent variables were one of the two PGS (PGS_EA_ and PGS_GI_). Whole brain measures (total surface area, mean cortical thickness, DWI_E_ or rsfMRI_E_) served as mediators. Finally, region‐specific multi‐mediator analyses were performed via elastic‐net regression for each combination of brain metric and PGS. Again, the I‐S‐T 2000 R sum score was the dependent and PGS the independent variable. Surface area, cortical thickness, DWI_Ei_ or rsfMRI_Ei_ of each HCPMMP area served as mediators.

Cortical surfaces of T1‐weighted images were reconstructed using FreeSurfer (http://surfer.nmr.mgh.harvard.edu, version 5.3.0), following an established protocol (Dale et al., 1999; Fischl et al., 1999). Pre‐processing included skull stripping, gray and white matter segmentation as well as reconstruction and inflation of the cortical surface. These steps were performed individually for each participant. Slice‐by‐slice quality control was performed and inaccuracies of automatic pre‐processing were edited manually. For the purpose of brain segmentation, we used the Human Connectome Project's multi‐modal parcellation (HCPMMP). Respective parcellation comprises 180 areas per hemisphere and is based on structural, functional, topographical, and connectivity data of healthy participants (Glasser et al., 2016). The original data provided by the Human Connectome Project were converted to annotation files matching the standard cortical surface in FreeSurfer called fsaverage. This fsaverage parcellation was transformed to each participant's individual cortical surface and converted to volumetric masks. Since macrostructure (Cox et al., 2019) as well as white matter connections (Genç et al., 2019) of subcortical areas have been shown to be associated with intelligence, we added subcortical areas to the DWI analysis. For this, eight subcortical gray matter structures per hemisphere were added to the parcellation (thalamus, caudate nucleus, putamen, pallidum, hippocampus, amygdala, accumbens area, ventral diencephalon) (Fischl et al., 2004). All masks were linearly transformed into the native spaces of the diffusion‐weighted images and used as landmarks for graph theoretical connectivity analyses (see Figure 1). Additionally, a white matter mask as well as six regions representing the four ventricles of the brain were delineated to serve as a nuisance variable for later BOLD signal analyses in terms of partial correlation analyses. For clarity, these partial correlations will be referred to as correlations in the rest of the manuscript. The subcortical areas were not used in the rsfMRI analysis, because the resulting brain areas are not optimal for resting‐state analysis due to being large and functionally heterogenous (Ma et al., 2022). We computed a mean value for each brain region by averaging values across the left and right hemispheres (e.g., the value for area V1 is the mean of L_V1 and R_V1), as we did not have any specific hypotheses with regard to hemispheric differences. This resulted in 180 for the analysis of surface area, cortical thickness and resting‐state fMRI, and 180 cortical and cortical and 8 subcortical areas for the DWI analysis. This was due to the literature being highly inconsistent. While some studies report a positive association of functional and structural asymmetries with intelligence (Barbey et al., 2012; Santarnecchi et al., 2015), others report a negative association (Moodie et al., 2020; O'Boyle et al., 2005; Yeo et al., 2016), no association (Ntolka & Papadatou‐Pastou, 2018; Papadatou‐Pastou & Tomprou, 2015) or different directions of association depending on the specific item (Everts et al., 2009; Gläscher et al., 2009). However, an exploratory hemispheric‐specific analysis can be found in Figures S4, S5 and Table S5.

#### 2.5.2 Analysis of diffusion‐weighted data

Diffusion tensor modelling and probabilistic fiber tractography were conducted using the FDT toolbox (https://fsl.fmrib.ox.ac.uk/fsl/fslwiki/FDT) in FSL version 5.0.9. (https://fsl.fmrib.ox.ac.uk/fsl/fslwiki), following the standard protocol (Behrens et al., 2003). Image pre‐processing included eddy currents correction and head motion correction. Additionally, the gradient directions of each volume were adjusted using the rotation parameters that were obtained from head motion correction. As described in the previous section, the 180 cortical and 8 subcortical regions from each hemisphere were transformed into the native space of the diffusion‐weighted images. Subsequently, these transformed regions were used as seed and target regions for probabilistic fiber tractography. To this end, we used a dual‐fiber model implemented in the latest version of BEDPOSTX (https://users.fmrib.ox.ac.uk/~moisesf/Bedpostx_GPU/). This model allows for the representation of two fiber orientations per voxel and thus enables the modelling of crossing fibers, which produces more reliable results compared to single‐fiber models (Behrens et al., 2007). The classification targets approach implemented in FDT was used to perform probabilistic fiber tracking (Genç et al., 2019). Five thousand tract‐following samples were generated at each voxel. The step length was 0.5 mm and the curvature threshold was 0.2 (only allowing for angles larger than 80 degrees). In order to quantify the connectivity between a seed voxel and a specific target region, the number of streamlines originating from the seed voxel and reaching the target region was determined. Subsequently, the overall connectivity between two brain regions was determined by calculating the sum of all streamlines proceeding from the seed to the target region and vice versa.

#### 2.5.3 Analysis of resting‐state data

Resting‐state data were pre‐processed using the FSL toolbox MELODIC. The first two volumes of each resting‐state scan were discarded. This was done to allow for signal equilibration. Afterwards, motion correction (reference volume = third image), slice timing correction, as well as high‐pass temporal frequency filtering (0.005 Hz) was applied. We applied 6 mm spatial smoothing. We also applied ICA‐AROMA protocols as described by Pruim et al. (2015) to correct for micro movements. Analogous to the analysis of the diffusion data, all brain regions were transformed into the native space of the resting‐state images for functional connectivity analysis. For each region, a mean resting‐state time course was calculated by averaging the time courses of all corresponding voxels. We computed partial correlations between the average time courses of all cortical regions while controlling for several nuisance variables, namely all six motion parameters as provided by MELODIC as well as average time courses extracted from white matter regions and ventricles (see 2.5.1. analysis of anatomical data) (Fraenz et al., 2021). We applied Fisher *z*‐transformation to all correlation values (Fisher, 1921) to ensure that they were normally distributed.

### 2.6 Graph metrics

Graph metrics were calculated using the Brain Connectivity Toolbox (Rubinov & Sporns, 2010) in combination with in‐house MATLAB code. DWI networks consisted of 376 nodes, including 360 cortical (180 in each hemisphere) and 16 subcortical regions (8 in each hemisphere). Resting‐state fMRI networks consisted of 360 cortical nodes (180 in each hemisphere). We employed Holm‐Bonferroni pruning with a threshold of 0 (*α* = .01, one tailed) as proposed by Ivković et al. (2012) since this approach circumvents some risks of applying a fixed threshold to a network, namely excluding viable connections when the threshold is set too high or including spurious connections if the threshold is set too low. Here, using the variance of all weights in the upper matrix triangle of all participants, every edge weight is tested to determine, if it is a spurious network connection or not. For example, a vector containing the edge weights of the edge between L_V1 and L_V2 of all participants is tested against zero. The edge is removed from the network if its edge weights do not differ significantly from zero. After doing this once for every edge, the procedure is repeated with the variance of all remaining weights in the upper triangle, to specifically test if this connection is crucial considering the whole network. This is done until the network does not contain any spurious connections anymore (Ivković et al., 2012). This pruning method was specifically chosen since intelligence is attributed to a widely distributed network all over the brain. Therefore, we wanted to test the importance of a connection within the whole network considering all edges in a network by using the joint variance of all network edges. By following this approach, 65,357 edges from the DWI network and 717 edges from the resting‐state network were removed. Two nodes (LH_H and RH_H) were removed from the resting‐state network completely, as they did not show any connections to other nodes after pruning. Using the Brain Connectivity Toolbox, we computed global efficiency (DWI_E_ and rsfMRI_E_), a graph metric used in previous studies investigating the association between network connectivity and cognitive performance (Kruschwitz et al., 2018; Ma et al., 2017). Global efficiency quantifies how efficiently the information can be transferred across the brain (Sporns et al., 2004). Large edge weights and small shortest path lengths typically lead to an increase in this metric. The shortest path is defined as the minimal number of edges it needs to connect a pair of nodes. The shortest path lengths between all pairs of nodes are comprised in the distance matrix *d*. This matrix can be created by calculating the inverse of the weighted adjacency matrix and running Dijksta's algorithm (Dijkstra, 1959). The global efficiency of one specific brain region is called nodal efficiency. It is calculated as the average inverse shortest path length between node *i* and all other nodes *j* within a network *G* (DWI_Ei_ and rsfMRI_Ei_). Calculations for global and nodal efficiency for DWI and rsfMRI were performed in an identical manner. The global efficiency of the entire network is the average inverse shortest path length between each pair of nodes within *G* (*E*):

$$E=\frac{1}{n}\;\sum_{i\in G}E_{i}=\frac{1}{n}\;\sum_{i\in G}\frac{\sum_{j\in G,j\neq i}d_{\mathrm{ij}}^{-1}}{n-1}$$

### 2.7 Statistical analysis

All statistical analyses were conducted in R Studio (1.3.1093) with R version 4.1.0 (2021‐05‐18). Data points were treated as outliers if they deviated more than three interquartile ranges from the respective variable's group mean (I‐S‐T 2000 R sum score, total surface area, mean cortical thickness, DWI or rsfMRI global efficiency). In such cases, all data from the corresponding participant were removed from analysis. No subjects were excluded from analyses concerning cortical surface area and cortical thickness (523 remaining subjects). Three subjects were excluded from analyses concerning DWI connectivity (520 remaining subjects) and one subject was excluded from analyses concerning rsfMRI (522 remaining).

#### 2.7.1 Partial correlations

We computed partial correlations between the I‐S‐T 2000 R score, two PGS (EA and GI), and several brain parameters (total surface area, mean cortical thickness, DWI global efficiency and rsfMRI global efficiency) using the *partial.cor* function included in the *RcmdrMisc* package. Age and sex were treated as confounding variables and regressed out.

#### 2.7.2 Global mediation model

We computed a mediation model using the *lavaan* package. The I‐S‐T 2000 R sum score served as the dependent variable, the two PGS (EA and GI) served as the independent variable. Mediators were surface area, cortical thickness, DWI global efficiency and rsfMRI global efficiency. Furthermore, we controlled for age, sex, and the first four principal components of the population stratification. Figure 1 (bottom half, middle box) shows a schematic depiction of a single mediation model. We used the robust maximum likelihood estimator *MLM* with robust standard errors and a Satorra‐Bentler scaled test statistic (Satorra & Bentler, 1994).

#### 2.7.3 Brain area‐specific mediation via elastic‐net regression

##### Mediation analysis by regularization

Following the computation of global mediation models, we investigated if a set of specific brain areas mediates the effect of PGS on I‐S‐T 2000 R. For this purpose, we employed *exploratory mediation analysis by regularization*, a tool developed to identify a subset of mediators from a large pool of potential mediators (Serang et al., 2017; Serang & Jacobucci, 2020). This approach does not use *p*‐values to determine the statistical significance of a mediator. Hence, it does not require a standard correction procedure for multiple comparisons (e.g., FDR or Bonferroni [Góngora et al., 2020]). Instead, it utilizes regularization such as the least absolute shrinkage operator (lasso), which puts a penalty on effect sizes. Here, small effects are pushed down to zero and only strong effects remain non‐zero. An in‐depth explanation of this approach is provided by Serang et al. (2017).

In short, all potential mediators are included in the model and the corresponding regression weights *a* and *b* are penalized (Ammerman et al., 2018). The penalty term lambda is determined using *k*‐fold cross‐validation, which is a mechanism to prevent overfitting. Here, the data is split into *k* subsets. One of those subsets is selected as the testing set while the rest of the data is used as the training‐set. This is done *k* times with every subset being used as the testing set once. The mediation effect of a mediator is calculated by multiplying the regression parameters *a* and *b*. If either parameter is regularized to zero, the mediation effect also becomes zero. If both *a* and *b* remain non‐zero after regularization, the mediation effect will be non‐zero as well. After this penalization procedure, all potential mediators with non‐zero mediation effects are selected as mediators. While this method is a good way of eliminating mediators with small effect sizes, it also brings the effect sizes of real mediators close to zero. In order to address this potential bias, the model is fit again without penalization. With a model that only includes the pre‐selected subset of mediators, unbiased effect sizes can be acquired (Serang & Jacobucci, 2020).

In this manuscript, we employed elastic‐net regression. Elastic‐net is another type of regularized regression that combines lasso and ridge regression (Zou & Hastie, 2005). The difference between ridge and lasso‐regression is that the lasso penalty can shrink a parameter to zero, whereas ridge regression can only asymptotically shrink a parameter towards zero. Thus, lasso is suitable for models in which a lot of variables are expected to have no or little effect on the dependent variable, while ridge regression is suitable for models in which most variables are expected to have a considerable effect on the dependent variable. Elastic‐net regression can be considered an ideal approach if one does not have clear expectations regarding every variable. In comparison to lasso regression, elastic‐net regression is also better at handling correlations between variables (Zou & Hastie, 2005), which was an important factor in our decision to choose elastic‐net over lasso regression. Regularized elastic‐net regression has already been successfully applied in a previous study investigating the association between fluid/crystallized intelligence and the microstructure of multiple white‐matter tracts (Góngora et al., 2020).

##### Alterations to xmed function and recalculation of effect sizes

We employed an altered version of the function provided by Serang and Jacobucci (2020). The modified code can be found at https://osf.io/2qamu/. First, we did not use the lambda sequence specified in the *xmed* function, but the default lambda sequence the *glmnet* package (Friedman et al., 2010) which *xmed* interfaces to. This was done because the lambda sequence specified by *xmed* leads to ceiling effects in the lambda parameters. Second, the original function does not account for the direct effect of the independent variable (PGS) on the dependent variable (I‐S‐T 2000 R score). Thus, we included the PGS as an independent variable in the regression of the mediators on the dependent variable (path *b*). This effect was not penalized.

Apart from investigating which brain areas mediate the relationship between PGS and intelligence, we were also interested in the direct effects of PGS on the brain and the direct effects of the brain on intelligence (see Figure 1, paths *a*,*b*). Thus, we followed a similar approach to identify variables exhibiting non‐zero effects within path *a* and path *b* regressions. The threshold for detecting non‐zero effects was set to 0.01. This was done because the mediation effects are the product of the regularized *a* and *b* parameters, which take values below 1. Hence, mediation effects are smaller compared to the regularized *a* and *b* parameters. Again, coefficients were re‐estimated with *lavaan* to avoid biased effect sizes.

**FIGURE 2:**
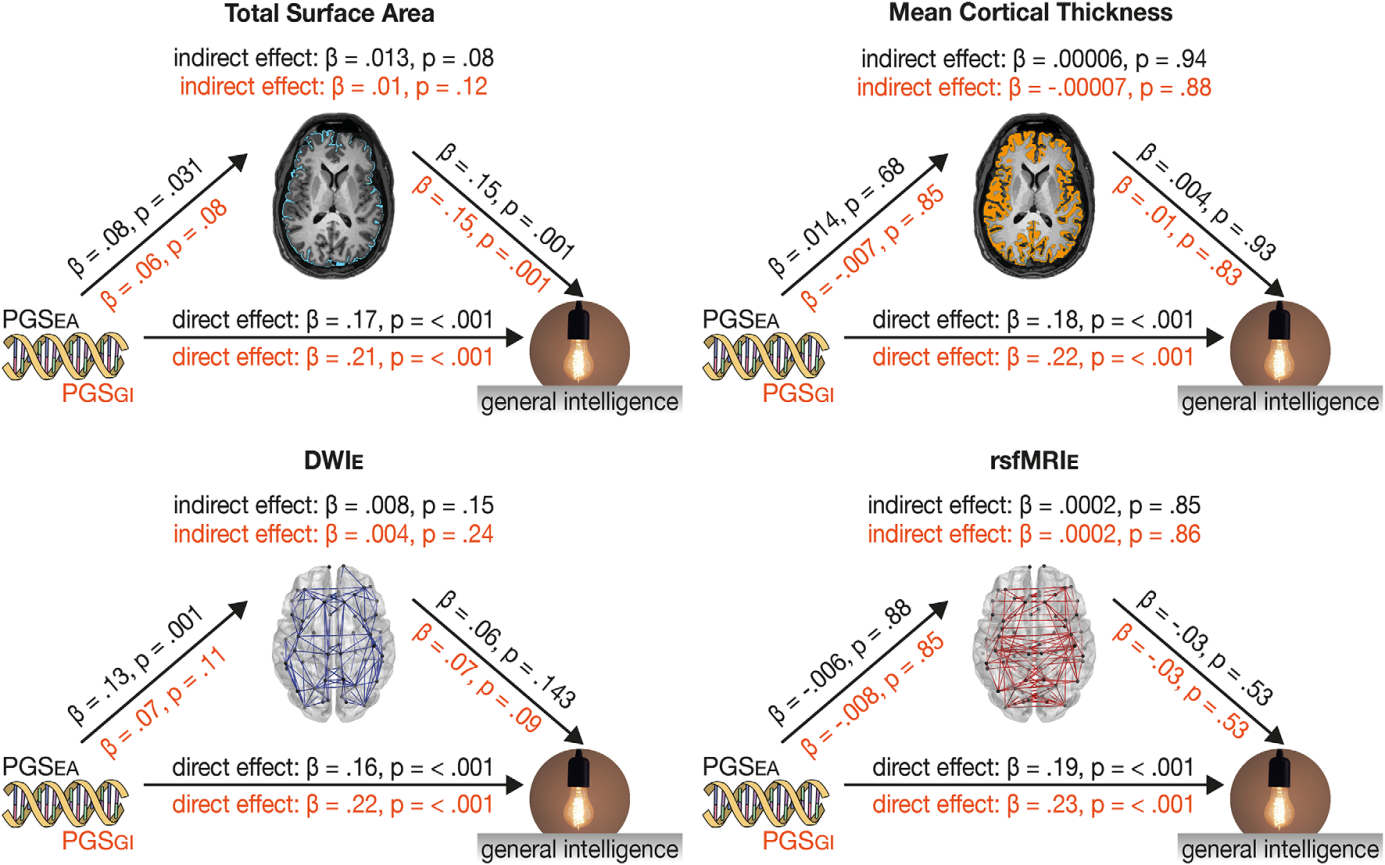
Results of the global mediation analysis. We used total surface area, mean cortical thickness, DWI_E_ and rsfMRI_E_ as mediators. In all cases, general intelligence, as measured by the I‐S‐T 2000 R sum score, served as the dependent variable. PGS_EA_ or PGS_GI_ served as independent variables. Effect sizes and *p*‐values are depicted in black (above the arrows) for analyses with PGS_EA_ and in orange (below the arrows) for analyses with PGS_GI_.

##### Specific mediation models

For the procedure described above, we used the *xmed* function from the *regsem* package (Ammerman et al., 2018; Serang et al., 2017; Serang & Jacobucci, 2020). All variables were standardized and residualized for sex, age and the first four principal components of population stratification. We computed 8 mediator models for all combinations of PGS (GI, EA) and brain parameters (surface area, cortical thickness, DWI nodal efficiency, rsfMRI nodal efficiency). This resulted in the following mediation models (independent variable–mediators–dependent variable): PGS_EA_–surface area–I‐S‐T‐2000 R score, PGS_EA_–cortical thickness– I‐S‐T‐2000 R score, PGS_EA_–DWI nodal efficiency– I‐S‐T‐2000 R score, PGS_EA_–rsfMRI nodal efficiency–I‐S‐T‐2000 R score, PGS_GI_–surface area–I‐S‐T‐2000 R score, PGS_GI_–cortical thickness–I‐S‐T‐2000 R score, PGS_GI_ –DWI nodal efficiency–I‐S‐T‐2000 R score, PGS_GI_–rsfMRI nodal efficiency–I‐S‐T‐2000 R score.

For all models, the number of cross‐validation folds was set to *k* = 80. The threshold for detecting non‐zero mediation effects was set to 0.001 and the type of regression was set to elastic‐net for all mediation models. PGS served as the independent variable and the I‐S‐T 2000 R sum score as the dependent variable. After the pruning procedure described above (see 2.6. Graph metrics), mediator models involving surface area and cortical thickness comprised 180 potential mediators each (180 cortical areas). The model involving DWI nodal efficiency comprised 188 potential mediators (180 cortical and 8 subcortical areas) and the model involving rsfMRI nodal efficiency comprised 179 potential mediators (179 cortical areas).

To investigate different dependencies and competitions between brain metrics, we calculated two exploratory models which comprise all brain metrics as mediators (727 mediators). The models were calculated as follows (independent variable – mediators – dependent variable): PGS_EA_ – surface area (180 cortical areas) + cortical thickness (180 cortical areas) + DWI nodal efficiency (188 cortical and subcortical areas) + rsfMRI nodal efficiency (179 cortical areas) – I‐S‐T 2000 R score, PGS_IQ_ – surface area (180 cortical areas) + cortical thickness (180 cortical areas) + DWI nodal efficiency (188 cortical and subcortical areas) + rsfMRI nodal efficiency (179 cortical areas) – I‐S‐T 2000 R score. Results are depicted in Figures S2 and S3. This analysis included participants that were not marked as an outlier for any of the brain metrics (n = 519).

### 2.8 Overlap of mediating areas and P‐FIT

Finally, we aimed to test whether the mediating brain areas overlapped with the P‐FIT network. It is important to note, that the P‐FIT network is based on Brodmann areas (BA). In the original version proposed by Jung and Haier (2007), the P‐FIT features a network of 14 BA. In an updated version by Basten et al. (2015) the network's composition was confirmed, but also extended with 5 additional BA. In order to compare the HCPMMP areas from our analyses with P‐FIT BA, we employed a cortical parcellation based on BA, which is included as an annotation file in FreeSurfer. This annotation file was converted to a volumetric segmentation matching the cortex of the fsaverage standard brain. The same was done to the HCPMMP annotation file. By means of an in‐house MATLAB program, the overlap between all HCPMMP and BA areas was calculated. An HCPMMP area was specified as being part of the P‐FIT network when it showed at least 80% overlap with one or more P‐FIT BA in both hemispheres. It was also specified as being P‐FIT when its activity was identified as being associated with fluid intelligence in both hemispheres in a recent meta‐analysis (Santarnecchi et al., 2017). This was true for 88 HCPMMP areas. Thus, this translation from BA to HCPMMP can be considered very liberal, as it classifies a large number of areas as being part of the P‐FIT network. Researchers who want to use this classification of a more detailed atlas or compare atlases in their studies can find a full list of all HCPMMP areas belonging to the P‐FIT with their respective BA and overlap in Table S1.

## 3 RESULTS

In preliminary analyses, to gain an overview of bivariate correlations and to compare our data with previously reported results, partial correlations were computed to test the associations between PGS and intelligence, PGS and whole brain properties, as well as whole brain properties and intelligence (see Table 1). Both PGS were significantly associated with the I‐S‐T 2000 R sum score (see Table 1) and total surface area. PGS_EA_ was also associated with DWI_E_. The I‐S‐T 2000 R sum score was associated with both total surface area and DWI_E_. Mean cortical thickness and rsfMRI_E_ were not associated with PGS or the I‐S‐T 2000 R sum score (see Table 1).

**TABLE 1:** Partial correlation coefficients (Pearson's r) between I‐S‐T 2000 R performance, PGS for education attainment (EA), general intelligence (GI) and brain properties.

	I‐S‐T 2000 R	SA	CT	DWI_E_	rsfMRI_E_
PGS_EA_	0.184***	0.106*	0.016	0.148***	0.006
PGS_GI_	0.235***	0.087*	−0.005	0.081	0.003
I‐S‐T 2000 R		0.149***	0.013	0.091*	−0.03

*Note*: Age and sex were used as controlling variables.

Abbreviations: CT, cortical thickness; DWIE, DWI‐network global efficiency; rsfMRIE, resting‐state network global efficiency; SA, surface Area.

* *p* < .05.

*** *p* < .001 (two‐tailed).

### 3.1 Global mediation analysis

Results of the global mediation analysis are shown in Figure 2. Even though preliminary analysis did not reveal an association between PGS and cortical thickness and rsfMRI_E_, we still investigated a potential mediation effect. This was done because the indirect effect cannot be concluded from *a* and *b* alone but is always the product *ab*, and statistical significance of *a* and *b* are not requirements for a mediation effect (Hayes, 2018; Zhao et al., 2010). PGS_EA_ was significantly associated with total surface area and DWI_E_. Total surface area and DWI_E_ were significantly associated with the I‐S‐T 2000 R sum score. However, none of the brain parameters turned out to be significant mediators in the effect of PGS on general intelligence on a whole brain level (all p > .08).

### 3.2 Brain area‐specific mediation

#### 3.2.1 Surface area

**FIGURE 3:**
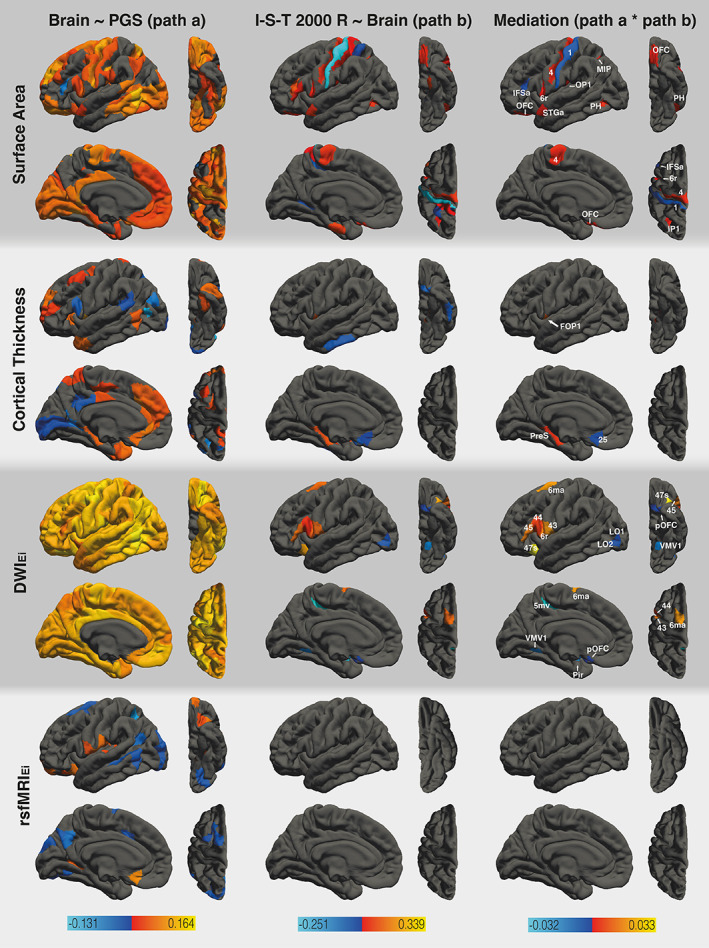
Results of the region‐specific multi‐mediator analysis via elastic net with PGS_EA_ as the dependent variable. The analysis employed the following mediators: surface area, cortical thickness, DWI_Ei_, and rsfMRI_Ei_ (from top to bottom). The figure shows the results from path *a* analysis, path *b* analysis, and the mediation effect (from left to right). Brain surfaces are shown in lateral, inferior, sagittal, and superior view (from left to right). Positive effects are depicted in red and yellow, negative effects are depicted in blue. Colored mediating areas are labeled according to the HCPMMP. Path *a* analysis of DWI_Ei_ also revealed positive associations between PGS_EA_ and eight subcortical areas. For a full list of areas and effect sizes see Tables S2 and S3.

Results of the region‐specific multi‐mediator analysis via elastic net showed that PGS_EA_ was associated with the surface area of the majority of HCPMMP areas (112 areas; Figure 3). All effects except one were positive, indicating that higher PGS_EA_ is associated with larger surface area (path *a*). Furthermore, the surface area of 18 brain areas in the parietal and frontal cortices was associated with the I‐S‐T 2000 R sum score (path *b*). Ten out of these brain areas mediated the effects of PGS_EA_ on general intelligence (a*b). HCPMMP areas 4 (primary motor cortex), 6r (premotor cortex), MIP, IP1 (intraparietal areas), OFC (orbital frontal cortex), OP1 (parietal operculum), STGa (anterior superior temporal gyrus), and PH (posterior temporal cortex) showed positive mediation effects. HCPMMP area 1 (somatosensory cortex) and IFSa (inferior frontal sulcus) showed negative mediation effects. Half of these areas were found to be part of the P‐FIT network (MIP, 6r, IFSa, PH, IP1).

**FIGURE 4:**
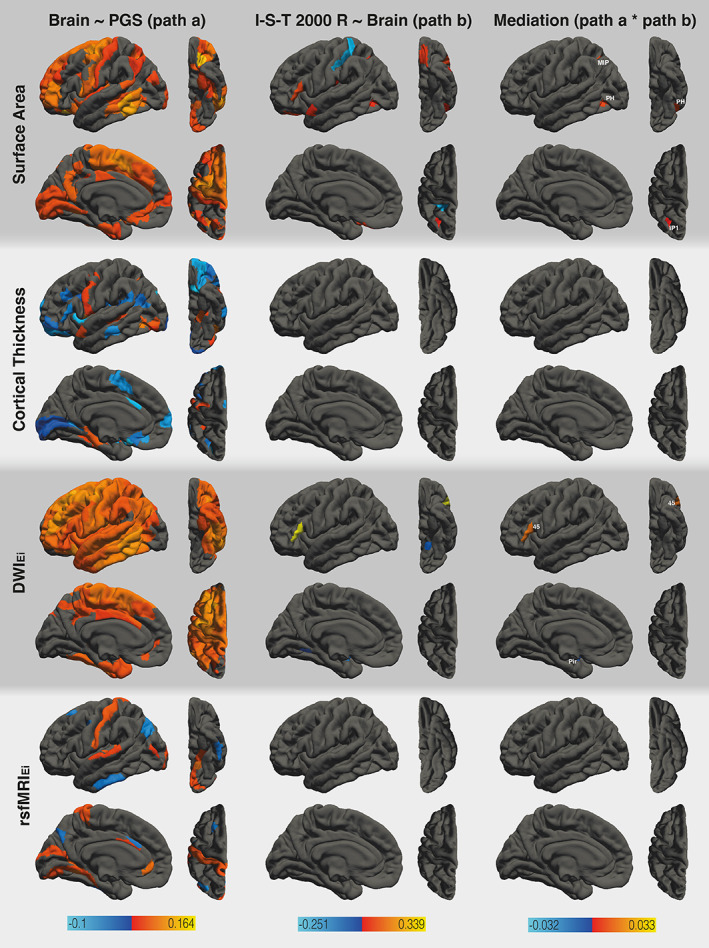
Results of the region‐specific multi‐mediator analysis via elastic net with PGS_GI_ as the dependent variable. The analysis employed the following mediators: surface area, cortical thickness, DWI_Ei_, and rsfMRI_Ei_ (from top to bottom). The figure shows the results from path *a* analysis, path *b* analysis, and the mediation effect (from left to right). Brain surfaces are shown in lateral, inferior, sagittal, and superior views (from left to right). Positive effects are depicted in red and yellow, negative effects are depicted in blue. Colored mediating areas are labeled according to the HCPMMP. Path *a* analysis of DWI_Ei_ also revealed positive associations between PGS_GI_ and six subcortical areas. For a full list of areas and effect sizes see Tables S2 and S3.

Similar results were obtained when PGS_GI_ was used as the predictor (see Figure 4). We found PGS_GI_ to be associated with the surface area of 87 brain areas distributed all over the cortex, with most areas largely matching (83%) those identified by the PGS_EA_ analysis. The surface area of eight areas was associated with general intelligence. Three of these areas mediated the effects of PGS_GI_ on general intelligence, namely HCPMMP areas MIP, IP1 (intraparietal areas), and PH (posterior temporal cortex). It is noteworthy, that all of these areas were identified as mediators in the PGS_EA_ analysis as well. All areas were found to be part of the P‐FIT network.

#### 3.2.2 Cortical thickness

PGS_EA_ was associated with cortical thickness in 39 brain areas, of which 26 (67%) exhibited positive effects and 13 (33%) exhibited negative effects. Seven cortical areas showed significant associations between cortical thickness and general intelligence. Three of these areas mediated the effects of PGS_EA_ on the I‐S‐T 2000 R sum score. HCPMMP areas FOP1 (frontal operculare area) and PreS (presubiculum) exhibited positive mediation effects, while area 25 (superior anterior cingulate cortex) exhibited a negative mediation effect. PGS_GI_ was associated with cortical thickness in 48 areas (21 positive associations and 27 negative associations) with limited overlap (23%) between the PGS_EA_ and PGS_GI_ analyses. The PGS_GI_ mediation model did not yield any areas which showed significant associations between cortical thickness and general intelligence. Consequently, no mediators for the effects of PGS_GI_ on general intelligence could be identified. None of the mediating areas were part of the P‐FIT network.

#### 3.2.3 DWI network efficiency

PGS_EA_ was positively associated with DWI_Ei_ in all cortical and subcortical areas (188). There were 12 areas in which DWI_Ei_ was associated with general intelligence. All of these areas were also mediators regarding the effects of PGS_EA_ on general intelligence. HCPMMP areas 6ma (anterior supplementary motor cortex), 6r (premotor cortex), 44, 45 (inferior frontal gyrus), 47 s (orbitofrontal cortex), and 43 (posterior opercular cortex) exhibited positive mediation effects. HCPMMP areas LO1, LO2 (lateral occipital cortex), 5mv (superior parietal cortex), VMV1 (ventromedial visual area), Pir (piriform cortex), and pOFC (posterior orbitofrontal complex) exhibited negative mediation effects. Half of these areas were found to be part of the P‐FIT network (LO1, LO2, 44, 45, 6r, 6ma). Similarly, PGS_GI_ was positively associated with DWI_Ei_ in 144 areas. DWI_Ei_ was associated with general intelligence in three areas and two of them, namely HCPMMP areas 45 (inferior frontal gyrus) and Pir (piriform cortex), were also found to be mediators regarding the effects of PGS_GI_ on general intelligence. These two areas were also identified as mediators in the PGS_EA_ analysis. HCPMMP area 45 was found to be part of the P‐FIT network.

#### 3.2.4 rsfMRI network efficiency

PGS_EA_ was associated with rsfMRI_Ei_ in 29 areas, with 13 (45%) showing a positive association. There were no areas that exhibited significant associations between rsfMRI_E_ and general intelligence or mediated the effects of PGS_EA_ on general intelligence. PGS_GI_ was associated with rsfMRI_Ei_ in 35 areas, with 18 (51%) of them showing positive associations. There were no areas that exhibited significant associations between rsfMRI_Ei_ and general intelligence or mediated the effects of PGS_GI_ on general intelligence.

Complete lists of HCPMMP areas and effect sizes can be found in Table S2 (path *a*), Table S3 (path *b*), and Table S4 (mediation).

### 3.3 Exploratory multimodal region‐specific multi‐mediator analysis

To investigate different dependencies and competitions between brain metrics, we calculated two exploratory models which comprise all brain metrics as mediators (728 mediators). The results are depicted in Figures S2 and S3. While this analysis revealed fewer mediators than the main analysis, the chosen mediators are the same. For PGS_EA_, the exploratory analysis revealed the surface area of IFSa, 4, MIP and IPS to act as mediators. Additionally, cortical thickness of FOP1 and PreS as well as DWI network efficiency of HCPMMP 45 were identified as mediators. For PGSGI it identified surface area of HCPMMP 4 as a mediator.

## 4 DISCUSSION

Genetic variability robustly predicts interindividual differences in intelligence, but it is still largely unknown which neurobiological intermediates are involved in the path from genetic disposition to phenotype. Hence, it was the aim of our study to conduct integrative analyses encompassing genome‐wide SNP variability, in‐depth brain imaging, and detailed measurement of cognitive abilities. By doing so, we were able to show that regional surface area and structural network efficiency are mediators of the relationship between genetic disposition and measured intelligence.

In line with other studies, PGS significantly predicted cognitive abilities. Furthermore, PGS were associated with morphological and connectivity brain measures of widely distributed cortical and subcortical regions, a finding which is in accordance with previously reported results showing genetic correlations between cognitive abilities and brain structure (Grasby et al., 2020). To further investigate which of these brain areas link genetic variation to differences in cognitive abilities, four brain properties on global and regional level were tested as putative mediators. rsfMRI was not associated with cognitive abilities, neither on a global scale nor on the level of brain regions. In case of cortical thickness, there was limited evidence of mediation effects. However, the surface area and structural connectivity of several brain areas were associated with intelligence and also identified as mediators.

With regard to surface area, we found ten brain regions that mediated the effects of PGS_EA_ on general intelligence. Respective areas were mainly located in the posterior parietal, posterior temporal, and superior frontal cortices. Three of these areas were also identified when PGS_GI_ was used as predictor and half of the mediating areas were part of the P‐FIT network (MIP, 6r, IFSa, PH, IP1). There were five brain areas outside of the P‐FIT network, namely the primary motor cortex (4), the primary somatosensory cortex (1), the orbitofrontal cortex (OFC), and the posterior part of the parietal operculum (OP1). The common observation that the volume or surface area of cortical gray matter is positively associated with intelligence is typically explained in the following way. Individuals with more cortical volume or surface area are likely to possess more neurons (Leuba & Kraftsik, 1994; Pakkenberg & Gundersen, 1997). A higher count in cortical neurons also indicates a higher number of synapses (Karbowski, 2007). Therefore, it is assumed that individuals with more cortical gray matter have more computational power to engage in problem solving and logical reasoning (Genç et al., 2018). Following this explanation, our results indicate that the SNPs associated with cognitive abilities may influence the gene expression related to neuron and synapse count within specific cortical areas. This in turn might influence intelligent thinking.

Our findings related to non‐P‐FIT areas are largely in line with the findings by Lett et al. (2020), who also found a mediating effect of surface area in parts of the primary motor cortex, the orbitofrontal cortex, and the parietal operculum. The orbitofrontal cortex and its interaction with the anterior cingulate cortex have been associated with decision making (Fatahi et al., 2020). The orbitofrontal cortex encodes the value of available choices based on past experiences. The anterior cingulate cortex is involved in a more “down‐stream” processing of decision consequences (Wallis & Kennerley, 2011). While the primary motor cortex is usually not associated with intelligence, its structural and functional properties have been found to change in accordance with verbal and non‐verbal intelligence in teenagers (Ramsden et al., 2011). The authors argue that this finding is indicative of an interrelation between cognitive and motor development (Wallis & Kennerley, 2011), which may also be one reason behind the association between motor skills, cognitive performance, and academic achievements (Syväoja et al., 2019; Trecroci et al., 2021). Although the primary somatosensory cortex was not identified as a mediator by Lett et al. (2020) or Elliott et al. (2019), a meta‐analysis revealed its functional properties to be associated with fluid intelligence (Santarnecchi et al., 2017).

Many biological theories of intelligence highlight the importance of efficient information exchange across the brain. Naturally, this task is heavily dependent on the structural quality of an extensive brain network. A neuronal circuitry associated with higher intelligence is thought to foster a more directed information processing along relevant areas within the network. Our findings support this assumption by showing that the structural nodal efficiency of twelve brain areas mediated the relationship between PGS_EA_ and general intelligence. Moreover, two of these brain areas were also identified in the PGS_GI_ analyses. It is noteworthy that half of the mediating areas are part of the P‐FIT network (6ma, 6r, 44, 45, LO1, LO2). The inferior frontal gyrus (45, 44), the premotor cortex (6r), and the anterior supplementary motor cortex (6ma) exhibited positive mediation effects. Parts of the lateral orbitofrontal cortex (LO1, LO2) exhibited negative mediating effects, which was due to negative associations between their structural connectivity and general intelligence. We also observed multiple mediators outside of the P‐FIT network. The ventromedial visual area (VMV1) and posterior orbitofrontal cortex (pOFC) exhibited negative mediation effects, which was due to negative associations between their nodal efficiency and general intelligence. The orbitofrontal cortex (47 s) and posterior opercular cortex (43) exhibited positive mediation effects. Functional properties of the right orbitofrontal cortex have been shown to be positively associated with fluid intelligence in a recent meta‐analysis (Santarnecchi et al., 2017). The posterior opercular cortex is part of the so‐called cingulo‐opercular network (Power & Petersen, 2013) which plays a critical role in intelligence according to the Network Neuroscience Theory (Barbey, 2018). This theory proposes that the neural basis of general intelligence is manifested in the dynamics of multiple brain‐wide modular networks. In other words, the Network Neuroscience Theory emphasizes that intelligence depends on the efficiency with which specific brain networks can be reorganized and adapted to a situation. It has to be noted that this theory is focused on the dynamic state of networks and is largely based on functional studies. Hence, it may not directly be applicable to white matter connectivity, even though functional networks have been proposed to arise from structural connectivity (Park & Friston, 2013). The Network Neuroscience Theory proposes that crystallized intelligence relies on easy‐to‐reach functional network states which in turn rely on strong connections between some highly connected brain areas. In contrast, fluid intelligence is supposed to rely on difficult‐to‐reach network states, which in turn rely on weak connections between networks. Weak connections giving rise to difficult‐to‐reach network states are located in the frontoparietal network and the cingulo‐opercular network (Barbey, 2018). For the most part, P‐FIT emerged from macrostructural studies. When looking at intelligence from a connectivity‐based perspective, as is done in Network Neuroscience Theory, it seems plausible that there are brain areas whose morphological properties are not related to intelligence, while their connectivity patterns are. Our results support this assumption by showing that a group of SNPs, identified by GWAS, is likely to influence the gene expression shaping the structural efficiency of specific areas from an extensive and intelligence‐related brain network.

Our results concerning the surface area and structural connectivity show that there are considerably more brain areas mediating the effect between PGS_EA_ and general intelligence than between PGS_GI_ and general intelligence. In all likelihood, this is due to the difference in discovery sample sizes of respective GWAS. PGS_EA_ was derived from a GWAS with a sample size of 1,131,881 individuals (Lee et al., 2018), whereas PGS_GI_ was derived from a GWAS with a sample size of 269,867 individuals (Savage et al., 2018). This results in the greater predictive power of PGS_EA_. While PGS_GI_ exhibited a stronger association with general intelligence in our sample compared to PGS_EA_, PGS_EA_ exhibited stronger associations with the analyzed brain properties (see Table 1). Some of the genes identified by Lee et al. (2018) (PGS_EA_) are highly expressed in the brain prenatally and thus influence the very early stages of brain development. Other genes show high expression both prenatally and postnatally. Functionally, the identified genes are involved in neurotransmitter secretion, the activation of ion channels and metabotropic receptors, as well as synaptic plasticity. Importantly, these genes are expressed in all parts of the nervous system and not limited to a certain set of brain areas. Our results are in line with this finding given that PGS_EA_ were associated with brain properties all over the cortex (Figures 3 and 4). However, since our analyses also included the phenotype, we were able to specify which parts of the brain are affected by intelligence‐related gene expression as identified by Lee et al. (2018). Importantly, this approach goes one step beyond investigating the genetic correlation between cognitive and brain phenotypes.

Whereas the direction of effect from genes to cognitive abilities and genes to brain structure is causal by definition (Plomin & von Stumm, 2018), it is conceivable that there is a bidirectional relationship between brain structure and cognitive abilities. Recent analyses used bidirectional latent causal variable and Mendelian randomization to assess the causal direction between human cortical structure, general intelligence, and educational attainment. They provide evidence for the influence of brain structure on general intelligence and educational attainment (Grasby et al., 2020). Our investigation includes both measured brain phenotype and detailed characterization of cognitive abilities and thus provides further evidence of causal processes between genetic variability and cognition through variation in brain structure and network connectivity.

In addition to our main analysis, we have also conducted an explanatory analysis in which the mediation model included all brain metrics as mediators at once (see Figures S2 and S3). Interestingly, the analysis yielded the same, albeit fewer, mediators as the main analysis, mainly regarding surface area (4, MIP, IFSa, IP1). It must be noted that this approach is rather exploratory, as previous studies have always looked at metrics separately and the number of mediators in this model is huge for our sample size. Thus, we suggest that future studies with possible larger sample sizes also employ joined mediation models for multiple brain metrics. This may give us insight into possible dependencies between brain metrics.

There are certain limitations to our study. First, PGS for educational attainment tends to overestimate genetically caused effects in non‐related samples (Abdellaoui & Verweij, 2021; Lee et al., 2018). Lee et al. (2018) showed that the predictive power of PGS declines as much as 40% when within‐family differences in educational attainment are taken into account, which is partly due to gene–environment correlations. The genes of parents also influence the rearing environment of their child, which results in a correlation between the environment and the genes a child inherits from their parents (Abdellaoui & Verweij, 2021). The effect of parental genes on rearing environment is demonstrated by the observation that even non‐shared genetic information of parents is predictive of a child's educational attainment (Kong et al., 2018). Thus, the predictive power of the PGS utilized in our study can in part be attributed to gene–environment correlations. Second, our functional connectivity analysis did not identify any regions that mediated the effects of PGS on general intelligence; or any brain area that was directly associated with general intelligence (see Figures 3 and 4). In order to compute nodal efficiency, we aggregated resting‐state data across the entire time span of our recordings. However, Network Neuroscience Theory argues that the crucial aspect of intelligence‐related functional networks is their dynamic flexibility (Barbey, 2018), which is not captured by the metrics we used. Hence, it is indeed conceivable that the flexibility of specific networks mediates the effects of genetic variation on general intelligence. Future studies using temporally high resolution rsfMRI and dynamic connectivity analyses should investigate the mediation effects of dynamic connectivity metrics.

This study is the first to investigate the mediating effects of multimodal, region‐specific brain properties on the association between genetic variation and intelligence. We show that the surface area and structural connectivity of frontal, sensory, motor, temporal, and anterior occipital brain regions provide a missing piece in the link between genetic variation and general intelligence. These findings are a crucial step forward in decoding the neurogenetic underpinnings of intelligence, as they identify specific regional networks that relate polygenic variation to intelligence.

## AUTHOR CONTRIBUTIONS

Erhan Genç, Robert Kumsta and Sebastian Ocklenburg conceived the project and supervised the experiments. Erhan Genç, Dorothea Metzen, Caroline Schlüter, Larissa Arning, Huu Phuc Nguyen, Onur Güntürkün, Robert Kumsta, and Sebastian Ocklenburg designed the project. Larissa Arning, Huu Phuc Nguyen, Fabian Streit, and Robert Kumsta planned and performed genetic experiments. Caroline Schlüter and Christoph Fraenz collected data. Erhan Genç, Caroline Schlüter, Larissa Arning, Dorothea Metzen, Manuel C. Voelkle, Christoph Fraenz, Robert Kumsta, and Sebastian Ocklenburg analyzed the data. Erhan Genç, Dorothea Metzen, Fabian Streit, Robert Kumsta and Sebastian Ocklenburg wrote the paper. All authors discussed the results and edited the manuscript.

## FUNDING INFORMATION

Funding for the research was provided by the Deutsche Forschungsgemeinschaft (DFG) grant number GU 227/16‐1 and SFB 1280 project A03 and F02 (project number: 316803389). FS acknowledges support from the German Federal Ministry of Education and Research (BMBF) through the ERA‐NET NEURON, “SynSchiz‐Linking synaptic dysfunction to disease mechanisms in schizophrenia‐a multilevel investigation” (01EW1810) grant.

## CONFLICT OF INTEREST STATEMENT

The authors declare that they have no competing interests.

## Supporting information


**Data S1:** Supplementary Information.

## Data Availability

The data and MATLAB code that support the findings of this study are available from the corresponding author upon reasonable request or can be downloaded from an Open Science Framework repository [https://osf.io/2qamu/].
