# Supplementary material for: Structural architecture and brain network efficiency link polygenic scores to intelligence
Source: Hum Brain Mapp. 2023 Apr 4;44(8):3359–76. doi: 10.1002/hbm.26286 (PMC10171514; doi:10.1002/hbm.26286)
Supplement: Supplementary file 1 — Data S1: Supplementary Information. [file HBM-44-3359-s001.pdf]

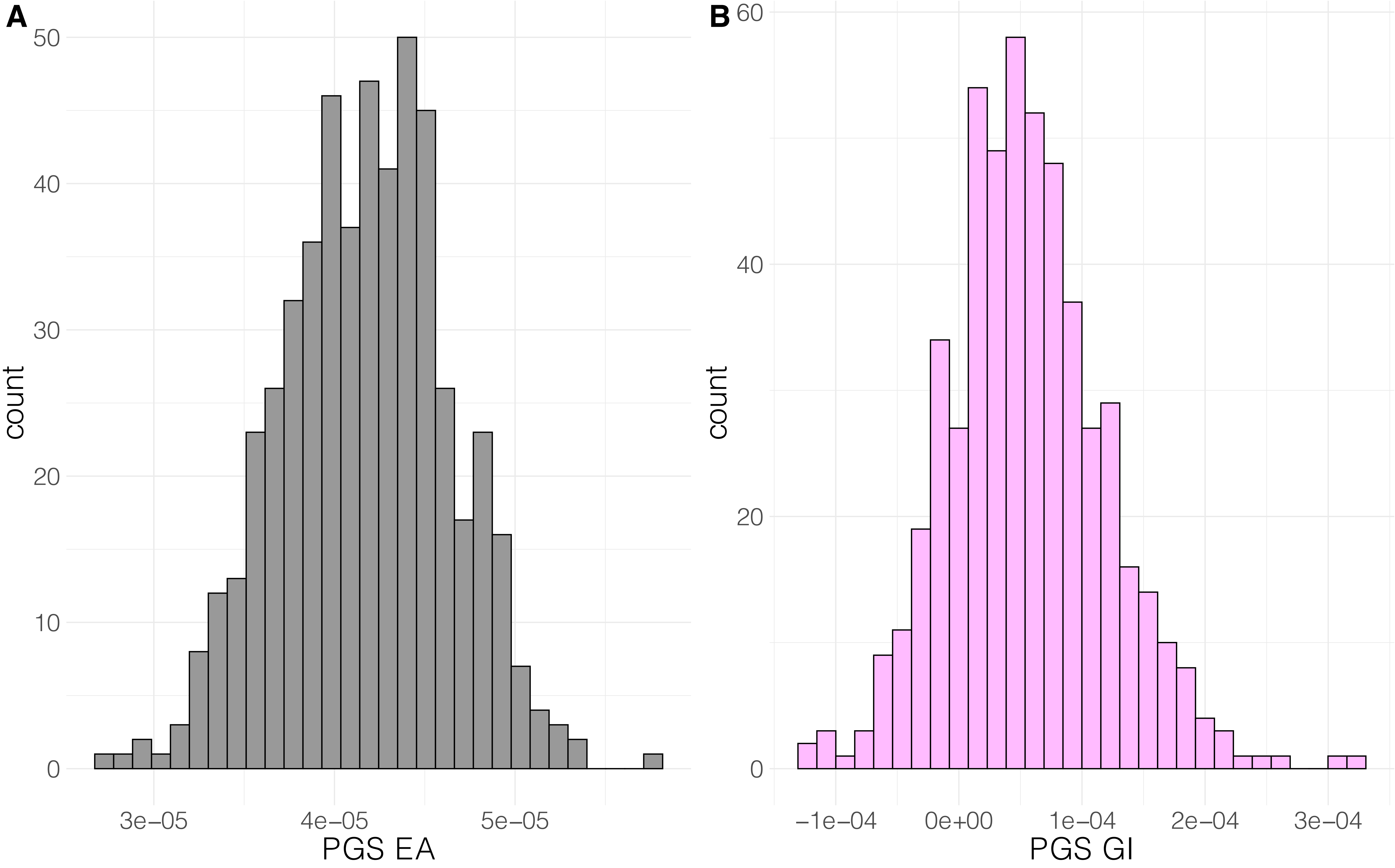

Supplementary Figure 1 A) Distribution of PGS for educational attainment in our sample B) Distribution of PGS for general intelligence in our sample.

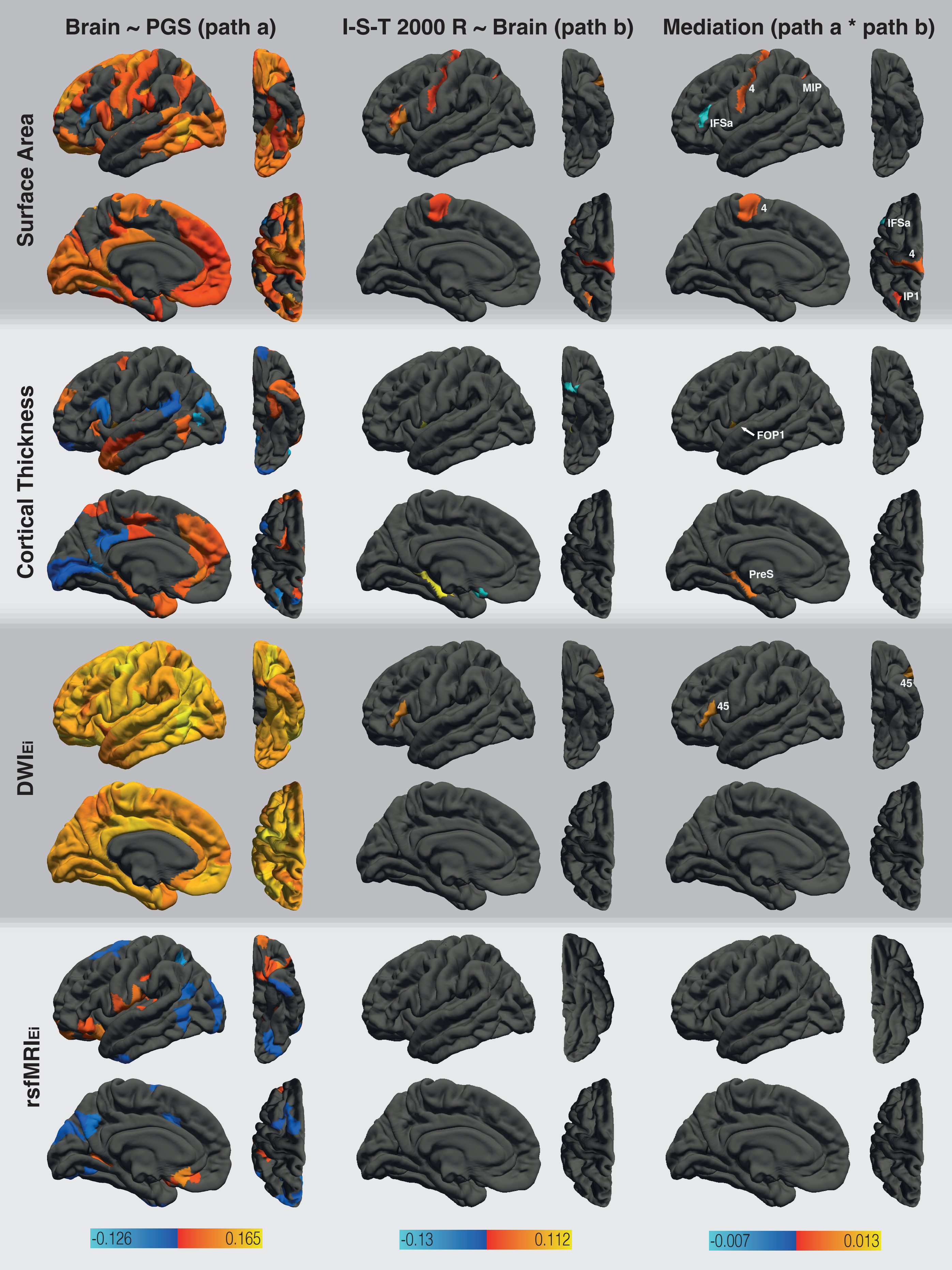

Supplementary Figure 2 Results of the multimodal region-specific multi-mediator analysis via elastic net with PGSEA as dependent variable. The analysis employed the following mediators: surface area, cortical thickness, DWIEi, and rsfMRIEi (from top to bottom). As opposed to the figures in the main manuscript, here one mediation analysis using all mediators at once was conducted. The figure shows the results from path a analysis, path b analysis, and the mediation effect (from left to right). Brain surfaces are shown in lateral, inferior, sagittal, and superior view (from left to right). Positive effects are depicted in red and yellow, negative effects are depicted in blue. Colored mediating areas are labeled according to the HCPMMP.

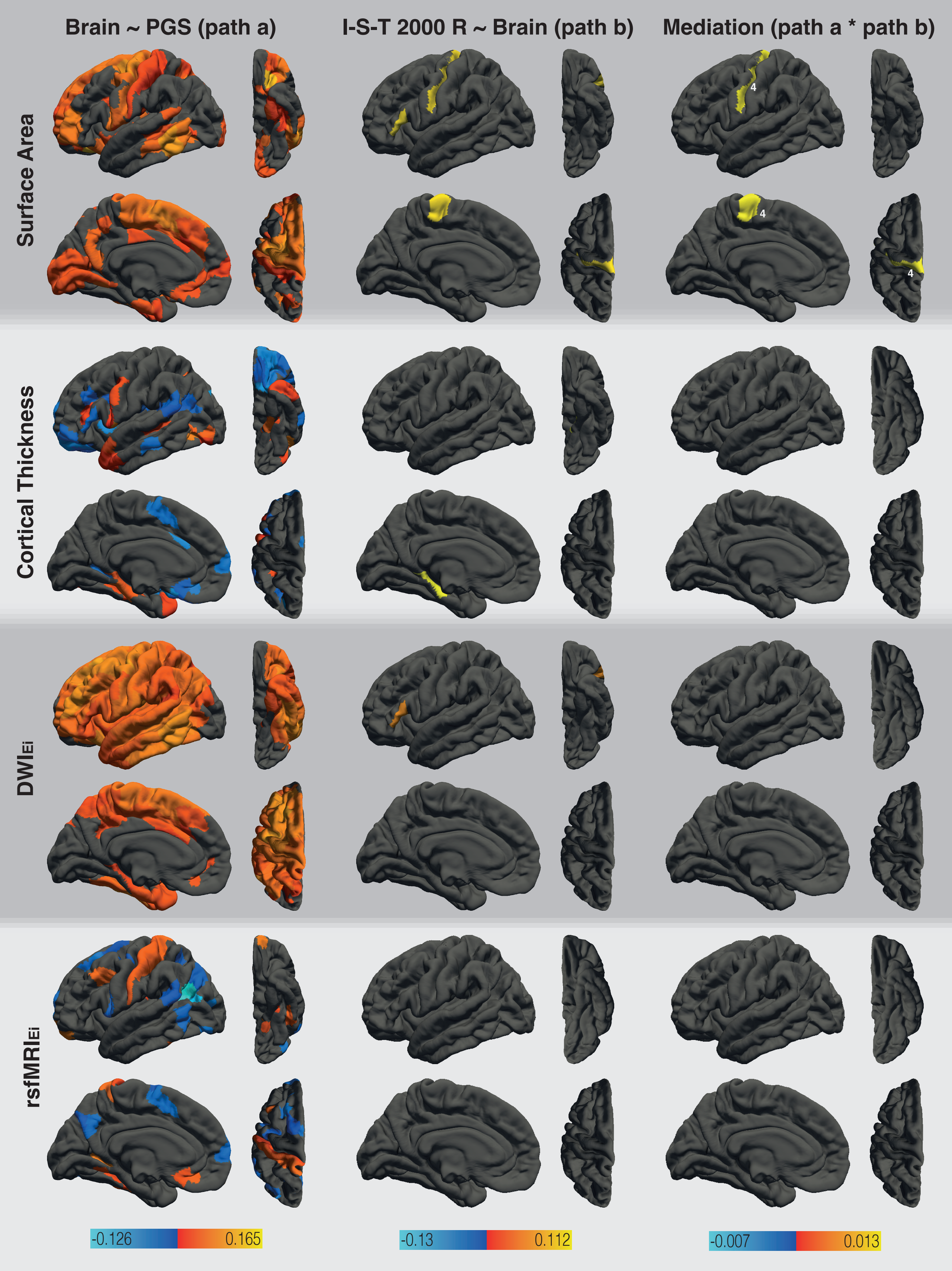

Supplementary Figure 3 Results of the multimodal region-specific multi-mediator analysis via elastic net with PGSGI as dependent variable. The analysis employed the following mediators: surface area, cortical thickness, DWIEi, and rsfMRIEi (from top to bottom). As opposed to the figures in the main manuscript, here one mediation analysis using all mediators at once was conducted. The figure shows the results from path a analysis, path b analysis, and the mediation effect (from left to right). Brain surfaces are shown in lateral, inferior, sagittal, and superior view (from left to right). Positive effects are depicted in red and yellow, negative effects are depicted in blue. Colored mediating areas are labeled according to the HCPMMP.

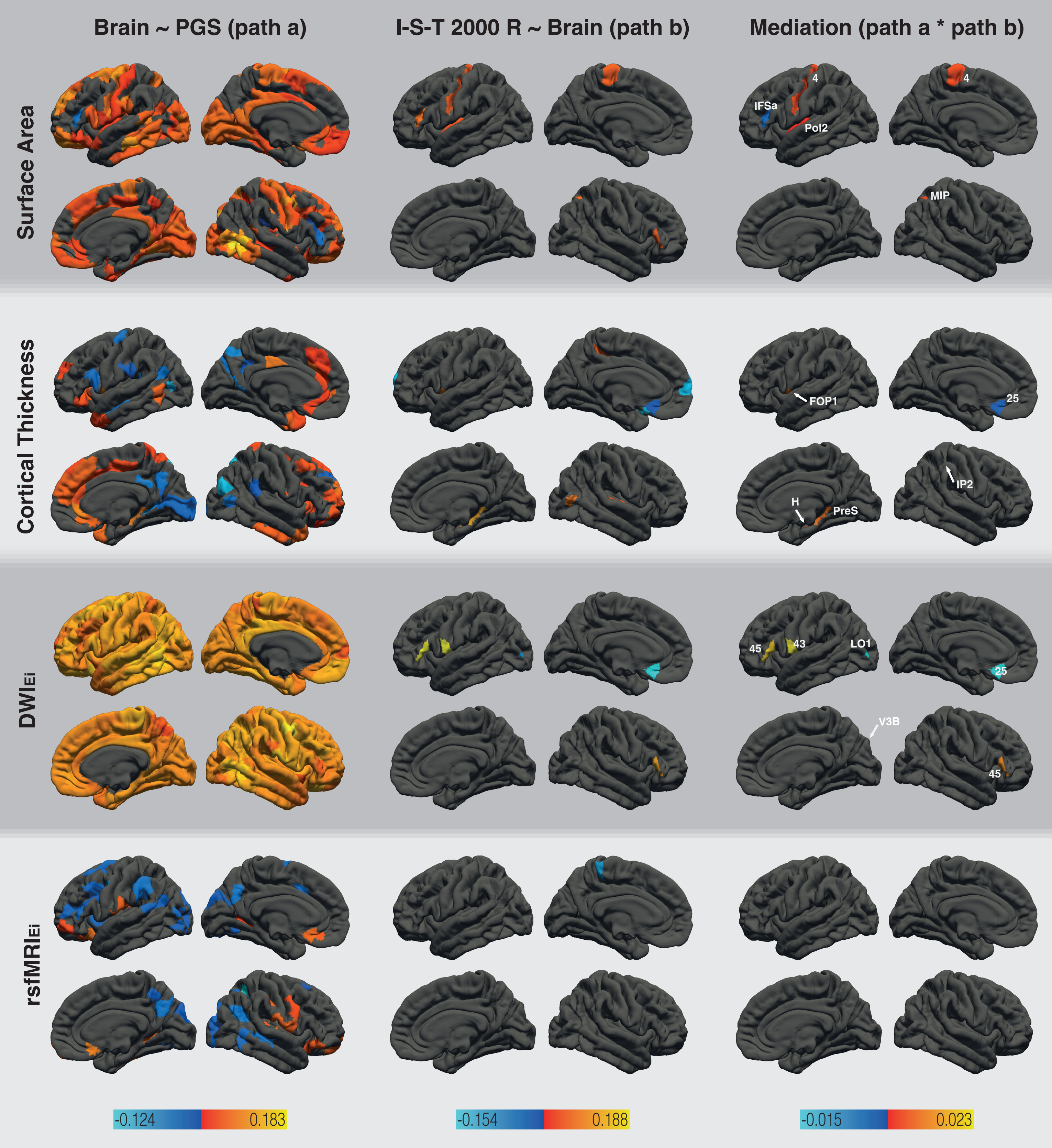

Supplementary Figure 4 Results of the multimodal region-specific multi-mediator analysis via elastic net with PGSEA as dependent variable for each hemisphere. The analysis employed the following mediators: surface area, cortical thickness, DWIEi, and rsfMRIEi (from top to bottom). As opposed to the figures in the main manuscript, the brain metrics were not avaraged over hemispheres. The figure shows the results from path a analysis, path b analysis, and the mediation effect (from left to right). Brain surfaces are shown for the left hemisphere and for the right hemisphere (from top to bottom). Positive effects are depicted in red and yellow, negative effects are depicted in blue. Colored mediating areas are labeled according to the HCPMMP.

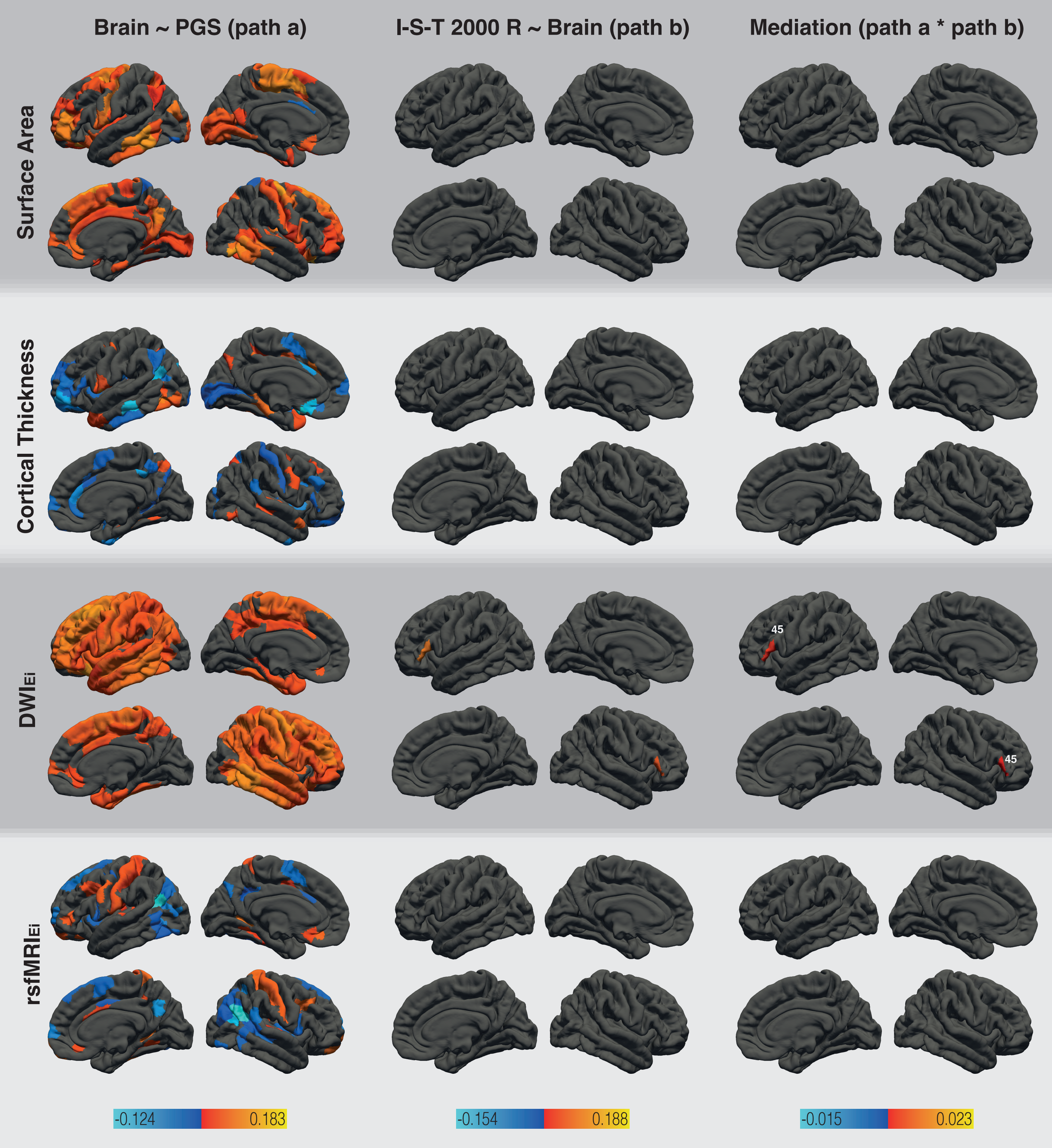

Supplementary Figure 5 Results of the multimodal region-specific multi-mediator analysis via elastic net with PGSGI as dependent variable for each hemisphere. The analysis employed the following mediators: surface area, cortical thickness, DWIEi, and rsfMRIEi (from top to bottom). As opposed to the figures in the main manuscript, the brain metrics were not avaraged over hemispheres. The figure shows the results from path a analysis, path b analysis, and the mediation effect (from left to right). Brain surfaces are shown for the left hemisphere and for the right hemisphere (from top to bottom). Positive effects are depicted in red and yellow, negative effects are depicted in blue. Colored mediating areas are labeled according to the HCPMMP.

Supplementary Table 1 **- HCPMMP that are part of the P-FIT network**. This table shows all HCPMMP that overlap with BAs of the P-FIT network at least 80% in both hemispheres. The first column states the HCPMMP, the second column states the overlapping BAs belonging to the P-FIT network, in both hemispheres respectively. Brackets show the percentage of overlap.

HCPMMP	BA (%)
10d	LH_10 (87.8), LH_9 (8.7), LH_32 (3.5), RH_10 (99), RH_9 (1)
10r	LH_32 (57.4), LH_10 (36.9), LH_24 (4.9), RH_10 (52.5), RH_32 (31.5)
23d	LH_24 (72.5), LH_31 (14.3), RH_24 (79.9), RH_31 (11.6)
24dv	LH_6 (55.7), LH_24 (44.3), RH_6 (70), RH_24 (23.3), RH_32 (3.1)
31a	LH_31 (88.8), RH_31 (97.7)
44	LH_44 (89.9), LH_45 (2.4), RH_44 (62), RH_45 (33.5)
45	LH_45 (63.3), LH_47 (18.9), LH_44 (2.4), RH_45 (61.6), RH_47 (34.1)
46	LH_46 (66.2), LH_9 (32.2), LH_8 (1.6), RH_46 (66.8), RH_9 (30.6), RH_8 (2.1)
6ma	LH_6 (87.4), RH_6 (95.9)
6r	LH_6 (78.4), LH_44 (16.1), RH_6 (45.4), RH_44 (44.8)
7PL	LH_7 (100), RH_7 (100)
7Pm	LH_7 (99.8), LH_31 (0.2), RH_7 (92.2), RH_31 (7.8)
8Ad	LH_8 (84.9), LH_6 (12.5), LH_9 (2.7), RH_8 (90.6), RH_9 (9.4)
8Av	LH_8 (66.9), LH_9 (23.2), LH_6 (9.9), RH_8 (56.3), RH_9 (39.9), RH_6 (3.8)
8BL	LH_8 (100), RH_8 (79.6), RH_9 (19.6)
8BM	LH_8 (62.8), LH_32 (20.5), LH_9 (15.1), RH_8 (82.8), RH_9 (11.5), RH_32 (4.2)
8C	LH_9 (42.1), LH_44 (34.1), LH_46 (19.7), RH_44 (38.3), RH_9 (34.8), RH_46 (26.1)
9-46d	LH_10 (49.1), LH_9 (39.9), LH_46 (10.3), RH_9 (54.9), RH_10 (43), RH_46 (2.1)
9a	LH_9 (94.5), LH_10 (5.5), RH_9 (79.6), RH_10 (20.4)
9m	LH_9 (62.6), LH_32 (22.2), LH_10 (7.3), RH_9 (69.4), RH_32 (15.6), RH_10 (13.6)
9p	LH_9 (86.4), LH_8 (13.6), RH_9 (100)
A1	LH_42 (96.3), RH_42 (77)
DVT	LH_19 (87.3), LH_7 (11.4), LH_18 (1.3), RH_19 (69.3), RH_7 (29.6), RH_31 (0.7)
FFC	LH_37 (96.4), LH_19 (3.6), RH_37 (98.4), RH_19 (1.6)
FST	LH_39 (38.4), LH_37 (27.6), LH_19 (21.8), RH_37 (86), RH_19 (12.5)
IFJa	LH_44 (100), RH_44 (89.6), RH_45 (10.4)
IFJp	LH_6 (61.5), LH_44 (37.6), LH_9 (0.9), RH_44 (75.2), RH_6 (24.8)
IFSa	LH_46 (58.1), LH_45 (41.7), LH_47 (0.3), RH_46 (54.9), RH_45 (27.5), RH_47 (17.6)
IFSp	LH_44 (39.4), LH_45 (37), LH_46 (22.9), RH_45 (76.9), RH_46 (20.2), RH_44 (2.4)
IP0	LH_19 (100), RH_19 (100)
IP1	LH_7 (74.1), LH_19 (25.9), RH_7 (53.9), RH_19 (45.5)
IP2	LH_7 (100), RH_7 (87.9), RH_40 (11.6)
IPS1	LH_19 (91.5), LH_7 (8.5), RH_19 (95.3), RH_7 (4.7)
LIPd	LH_7 (100), RH_7 (100)
LIPv	LH_7 (100), RH_7 (100)
LO1	LH_19 (100), RH_19 (100)
LO2	LH_19 (100), RH_19 (96.6), RH_37 (3.4)
LO3	LH_19 (98.6), LH_39 (1.4), RH_19 (100)
MIP	LH_7 (91.5), LH_19 (8.5), RH_7 (69.3), RH_19 (30.7)
MST	LH_39 (65.6), LH_19 (34.4), RH_19 (69.4), RH_39 (26.9), RH_37 (0.5)

MT	LH_19 (53.7), LH_39 (46.3), RH_19 (97.3), RH_39 (2.7)
PEF	LH_6 (100), RH_6 (99.3), RH_9 (0.8)
PF	LH_40 (84.6), LH_7 (15), RH_40 (94.3)
PFm	LH_7 (54.3), LH_40 (43.4), LH_39 (1.4), RH_40 (70.5), RH_7 (20.8), RH_39 (8.4)
PGi	LH_39 (62.8), LH_40 (37.2), RH_39 (90.1), RH_40 (9.7), RH_19 (0.2)
PGp	LH_19 (85.7), LH_39 (13.7), RH_19 (99.9), RH_39 (0.1)
PGs	LH_39 (52.4), LH_7 (27.8), LH_19 (16.6), RH_39 (45.7), RH_19 (27.9), RH_7 (17)
PHA2	LH_20 (100), RH_20 (95.6)
PHA3	LH_20 (74.3), LH_37 (25.7), RH_20 (77.1), RH_37 (22.1)
PH	LH_37 (98.4), LH_19 (1.6), RH_37 (99.9), RH_19 (0.1)
PIT	LH_19 (98.2), LH_37 (1.8), RH_19 (89.1), RH_37 (10.9)
POS2	LH_7 (40.8), LH_31 (29.4), LH_19 (28.5), RH_31 (55.8), RH_7 (28.4), RH_19 (7.1)
SCEF	LH_6 (77.2), LH_8 (8.6), LH_32 (7), RH_6 (79.9), RH_8 (18.6)
SFL	LH_6 (72.8), LH_8 (27.2), RH_6 (55.1), RH_8 (44.9)
TE2a	LH_21 (75.8), LH_20 (23.5), RH_21 (71.3), RH_20 (22.6), RH_37 (4.7)
TE2p	LH_37 (60.8), LH_20 (22.5), LH_21 (15), RH_37 (87.1), RH_20 (10.5)
TF	LH_20 (88.4), LH_37 (11.6), RH_20 (74), RH_37 (26)
TPOJ2	LH_39 (100), RH_39 (78.8), RH_37 (6.5)
TPOJ3	LH_39 (96.9), LH_19 (3.1), RH_39 (66), RH_19 (34)
V1	LH_18 (33.8), RH_18 (58.9), RH_19 (1.7)
V3A	LH_18 (97.6), LH_19 (2.4), RH_18 (95.6), RH_19 (4.4)
V3B	LH_19 (97.1), LH_18 (2.9), RH_18 (56.6), RH_19 (43.4)
V3CD	LH_19 (100), RH_19 (73.4), RH_18 (26.6)
V3	LH_18 (60.2), LH_19 (39.8), RH_18 (55.5), RH_19 (44.5)
V4	LH_19 (71.7), LH_18 (28.3), RH_19 (72.8), RH_18 (27.2)
V4t	LH_19 (100), RH_19 (97.6), RH_37 (2.4)
V6A	LH_19 (69.3), LH_18 (30.7), RH_19 (97), RH_7 (3)
V6	LH_18 (58.4), LH_19 (40.9), RH_19 (87.2), RH_18 (12.3)
V7	LH_19 (71.9), LH_18 (28.1), RH_19 (55.5), RH_18 (44.5)
V8	LH_19 (74.5), LH_37 (22.9), RH_19 (83.8), RH_37 (14.8)
VIP	LH_7 (100), RH_7 (100)
VMV2	LH_37 (50.3), LH_19 (29.9), LH_20 (16.6), RH_20 (53.4), RH_19 (24.7), RH_37 (20.3)
VMV3	LH_37 (59.6), LH_19 (40.4), RH_37 (64), RH_19 (34.5)
VVC	LH_37 (88.4), LH_20 (11.3), LH_19 (0.3), RH_37 (92.6), RH_20 (6.5), RH_19 (0.9)
a24pr	LH_24 (100), RH_24 (100)
a32pr	LH_32 (66.4), LH_24 (33.6), RH_32 (63.5), RH_24 (35.4), RH_8 (1.1)
a9-46v	LH_10 (83.6), LH_46 (16.4), RH_10 (86.9), RH_46 (13.1)
d32	LH_32 (86.9), LH_24 (6.6), RH_32 (73.8), RH_9 (21.5), RH_24 (4.2)
i6-8	LH_6 (94.2), LH_8 (5.8), RH_6 (52.3), RH_8 (47.7)
p10p	LH_10 (100), RH_10 (99.1)
p24	LH_24 (96.7), RH_24 (92.5)
p24pr	LH_24 (100), RH_24 (100)
p32	LH_32 (63.4), LH_24 (36.4), RH_32 (65.9), RH_24 (31.6), RH_10 (2.5)
p32pr	LH_24 (56.6), LH_32 (35.7), LH_6 (3.5), RH_32 (49.4), RH_24 (34.3), RH_8 (10.3)
p47r	LH_46 (56), LH_47 (24.4), LH_10 (18.4), RH_47 (52.8), RH_10 (24), RH_46 (18.1)
p9-46v	LH_46 (92.7), LH_9 (7.3), RH_46 (99.8), RH_9 (0.2)
s32	LH_24 (71.8), LH_32 (26.8), RH_32 (64.6), RH_24 (29)
s6-8	LH_6 (56.2), LH_8 (43.8), RH_8 (59.1), RH_6 (40.9)

Supplementary Table 2 - **Path a coefficients**. Coefficients from the a path (PGS - brain) of the mediation analysis after re-estimation with lavaan. Analyses were conducted with two different PGS (EA, GI). Results from the surface area (SA) analysis are depicted in green, results from the cortical thickness (CT) analysis in blue, results from the DWI analysis in red, results from the rsfMRI anaylsis in yellow.

PGS-EA		PGS-GI		PGS-EA		PGS-GI		PGS-EA		PGS-GI		PGS-EA		PGS-GI	
UV	a	UV	a	UV	a	UV	a	UV	a	UV	a	UV	a	UV	a
SA_V1	0.076	SA_V1	0.055	CT_V1	-0.042	CT_V1	-0.039	DWI_V1	0.112	DWI_MST	0.071	rsfMRI_V3	-0.050	rsfMRI_3b	0.052
SA_MST	0.100	SA_V2	0.053	CT_MT	-0.099	CT_FEF	0.048	DWI_MST	0.143	DWI_4	0.083	rsfMRI_V4	-0.054	rsfMRI_POS2	-0.048
SA_V6	0.064	SA_4	0.079	CT_PSL	-0.042	CT_PEF	0.045	DWI_V6	0.109	DWI_3b	0.079	rsfMRI_V3A	-0.052	rsfMRI_PIT	-0.062
SA_V2	0.086	SA_3b	0.072	CT_STV	-0.050	CT_V7	-0.085	DWI_V2	0.115	DWI_FEF	0.112	rsfMRI_POS2	-0.057	rsfMRI_STV	-0.049
SA_V3	0.077	SA_FEF	0.106	CT_POS1	-0.072	CT_LO2	0.061	DWI_V3	0.114	DWI_PEF	0.081	rsfMRI_V7	-0.056	rsfMRI_7m	-0.064
SA_V4	0.089	SA_PEF	0.079	CT_23d	0.057	CT_PIT	0.041	DWI_V4	0.113	DWI_55b	0.096	rsfMRI_7m	-0.067	rsfMRI_5L	0.074
SA_4	0.094	SA_55b	0.082	CT_d23ab	-0.052	CT_PSL	-0.056	DWI_V8	0.113	DWI_IPS1	0.046	rsfMRI_6ma	-0.053	rsfMRI_SCEF	-0.064
SA_3b	0.083	SA_IPS1	0.050	CT_5L	0.044	CT_SCEF	-0.066	DWI_4	0.133	DWI_FFC	0.080	rsfMRI_LIPv	-0.092	rsfMRI_LIPv	-0.051
SA_FEF	0.083	SA_SFL	0.084	CT_24dd	0.046	CT_MIP	0.057	DWI_3b	0.126	DWI_MT	0.059	rsfMRI_p32pr	-0.051	rsfMRI_1	0.066
SA_PEF	0.101	SA_PCV	0.060	CT_7Am	0.048	CT_6v	0.047	DWI_FEF	0.158	DWI_A1	0.068	rsfMRI_10pp	0.076	rsfMRI_2	0.065
SA_55b	0.058	SA_STV	0.062	CT_7PL	-0.061	CT_a24pr	-0.077	DWI_PEF	0.134	DWI_SFL	0.082	rsfMRI_13l	0.055	rsfMRI_8Ad	-0.064
SA_V3A	0.057	SA_7m	0.067	CT_a24	0.087	CT_47m	-0.050	DWI_55b	0.156	DWI_PCV	0.060	rsfMRI_47s	0.083	rsfMRI_10d	-0.062
SA_RSC	0.102	SA_POS1	0.068	CT_d32	0.069	CT_10d	-0.068	DWI_V3A	0.124	DWI_STV	0.070	rsfMRI_LIPd	-0.068	rsfMRI_8C	0.073
SA_V7	0.093	SA_23d	0.053	CT_8BM	0.068	CT_45	-0.060	DWI_RSC	0.128	DWI_7Pm	0.054	rsfMRI_i6.8	-0.043	rsfMRI_6r	-0.054
SA_IPS1	0.064	SA_v23ab	0.069	CT_p32	0.063	CT_a47r	-0.051	DWI_POS2	0.099	DWI_23d	0.043	rsfMRI_s6.8	-0.057	rsfMRI_10pp	0.083
SA_FFC	0.056	SA_31pv	0.044	CT_8Av	0.041	CT_IFJa	-0.047	DWI_V7	0.135	DWI_31pv	0.060	rsfMRI_43	0.061	rsfMRI_13l	0.057
SA_V3B	0.066	SA_24dd	0.077	CT_9m	0.060	CT_p946v	-0.055	DWI_IPS1	0.132	DWI_5m	0.058	rsfMRI_OP4	0.081	rsfMRI_s6.8	-0.069
SA_LO1	0.095	SA_24dv	0.066	CT_44	-0.058	CT_10pp	-0.072	DWI_FFC	0.130	DWI_5mv	0.055	rsfMRI_OP1	0.069	rsfMRI_OP4	0.048
SA_LO2	0.081	SA_SCEF	0.098	CT_a946v	0.041	CT_11l	-0.074	DWI_V3B	0.103	DWI_23c	0.059	rsfMRI_OP2.3	0.067	rsfMRI_OP1	0.041
SA_PIT	0.068	SA_6ma	0.079	CT_9a	0.079	CT_13l	-0.068	DWI_LO1	0.136	DWI_5L	0.068	rsfMRI_52	0.055	rsfMRI_OP2.3	0.050
SA_MT	0.086	SA_7PL	0.063	CT_a10p	0.059	CT_43	0.043	DWI_LO2	0.108	DWI_24dd	0.061	rsfMRI_ProS	0.066	rsfMRI_FOP4	-0.051
SA_SFL	0.079	SA_LIPv	0.062	CT_6a	0.054	CT_OP1	-0.057	DWI_PIT	0.105	DWI_24dv	0.064	rsfMRI_STSvp	-0.052	rsfMRI_FOP2	0.053
SA_STV	0.088	SA_VIP	0.045	CT_RI	-0.060	CT_OP23	-0.058	DWI_MT	0.141	DWI_7AL	0.060	rsfMRI_PHT	-0.052	rsfMRI_ProS	0.091
SA_POS1	0.068	SA_MIP	0.083	CT_AVI	0.074	CT_RI	-0.048	DWI_A1	0.128	DWI_SCEF	0.082	rsfMRI_PGi	-0.065	rsfMRI_PHA3	0.048
SA_23d	0.083	SA_1	0.044	CT_FOP1	0.115	CT_MI	-0.076	DWI_PSL	0.105	DWI_6ma	0.091	rsfMRI_PHA2	0.060	rsfMRI_TE2p	0.061
SA_v23ab	0.084	SA_3a	0.073	CT_FOP3	0.069	CT_Pir	-0.071	DWI_SFL	0.116	DWI_7PC	0.064	rsfMRI_V3CD	-0.054	rsfMRI_PHT	-0.057
SA_d23ab	0.086	SA_6d	0.095	CT_PreS	0.069	CT_FOP1	0.096	DWI_PCV	0.092	DWI_LIPv	0.075	rsfMRI_25	0.096	rsfMRI_TPOJ3	-0.097
SA_31pv	0.055	SA_6mp	0.080	CT_ProS	-0.070	CT_FOP3	0.041	DWI_STV	0.138	DWI_VIP	0.071	rsfMRI_MBelt	0.080	rsfMRI_PGp	-0.048
SA_23c	0.046	SA_p32pr	0.069	CT_PeEc	0.059	CT_FOP2	0.064	DWI_7Pm	0.088	DWI_MIP	0.063	rsfMRI_LBelt	0.052	rsfMRI_PGi	-0.130
SA_6ma	0.093	SA_a24	0.056	CT_TGd	0.075	CT_AIP	0.046	DWI_7m	0.110	DWI_1	0.079			rsfMRI_PGs	-0.057
SA_7PL	0.080	SA_d32	0.062	CT_PHT	0.066	CT_PreS	0.064	DWI_POS1	0.115	DWI_2	0.074			rsfMRI_VMV1	0.059
SA_LIPv	0.081	SA_8BM	0.059	CT_PGp	-0.071	CT_H	0.056	DWI_23d	0.140	DWI_3a	0.086			rsfMRI_PHA2	0.042
SA_VIP	0.117	SA_p32	0.054	CT_IP1	-0.077	CT_ProS	-0.064	DWI_v23ab	0.122	DWI_6d	0.097			rsfMRI_V3CD	-0.068
SA_MIP	0.076	SA_47m	0.085	CT_31pd	-0.047	CT_STGa	0.053	DWI_d23ab	0.135	DWI_6mp	0.072			rsfMRI_25	0.052
SA_1	0.059	SA_8Ad	0.064	CT_25	0.075	CT_PHA1	0.065	DWI_31pv	0.144	DWI_6v	0.060			rsfMRI_s32	0.064
SA_3a	0.114	SA_8BL	0.081	CT_FOP5	0.078	CT_PHA3	0.049	DWI_5m	0.106	DWI_p24pr	0.054				
SA_6d	0.116	SA_9p	0.073	CT_p10p	0.043	CT_STSdp	0.051	DWI_5mv	0.125	DWI_33pr	0.043				
SA_6mp	0.066	SA_10d	0.046	CT_a32pr	0.091	CT_PH	0.086	DWI_23c	0.112	DWI_a24pr	0.065				
SA_6v	0.062	SA_a47r	0.081	CT_p24	0.067	CT_TPOJ3	-0.055	DWI_5L	0.122	DWI_p32pr	0.062				
SA_a24	0.062	SA_6r	0.083			CT_IP1	-0.054	DWI_24dd	0.117	DWI_8BM	0.055				
SA_d32	0.049	SA_IFJa	0.051			CT_PGi	-0.052	DWI_24dv	0.108	DWI_p32	0.051				
SA_8BM	0.050	SA_IFJp	0.068			CT_FST	0.060	DWI_7AL	0.088	DWI_47m	0.086				
SA_p32	0.057	SA_46	0.041			CT_VMV2	0.049	DWI_SCEF	0.105	DWI_8Av	0.100				
SA_10r	0.047	SA_a946v	0.092			CT_25	-0.054	DWI_6ma	0.140	DWI_8Ad	0.104				
SA_47m	0.086	SA_946d	0.053			CT_s32	-0.065	DWI_7Am	0.085	DWI_8BL	0.084				
SA_8Ad	0.064	SA_9a	0.061			CT_pOFC	-0.082	DWI_7PL	0.116	DWI_9p	0.096				
SA_9m	0.044	SA_a10p	0.062			CT_TE1m	-0.062	DWI_7PC	0.098	DWI_8C	0.078				
SA_8BL	0.075	SA_11l	0.054			CT_V2	-0.003	DWI_LIPv	0.124	DWI_44	0.065				
SA_9p	0.103	SA_13l	0.132					DWI_VIP	0.133	DWI_45	0.054				
SA_10d	0.059	SA_47s	0.076					DWI_MIP	0.135	DWI_47l	0.057				
SA_44	0.051	SA_6a	0.104					DWI_1	0.125	DWI_a47r	0.074				
SA_a47r	0.081	SA_i68	0.088					DWI_2	0.129	DWI_6r	0.066				
SA_6r	0.067	SA_s68	0.084					DWI_3a	0.134	DWI_IFJa	0.075				
SA_IFJp	0.062	SA_43	0.072					DWI_6d	0.147	DWI_IFJp	0.077				
SA_IFSa	-0.073	SA_OP1	0.057					DWI_6mp	0.117	DWI_IFSp	0.074				
SA_46	0.053	SA_PoI2	0.043					DWI_6v	0.134	DWI_IFSa	0.078				
SA_946d	0.071	SA_TA2	0.060					DWI_p24pr	0.118	DWI_p946v	0.092				
SA_9a	0.119	SA_AAIC	0.060					DWI_33pr	0.127	DWI_46	0.103				
SA_10v	0.057	SA_FOP1	0.057					DWI_a24pr	0.127	DWI_a946v	0.102				
SA_a10p	0.071	SA_FOP3	0.079					DWI_p32pr	0.122	DWI_946d	0.106				
SA_10pp	0.107	SA_FOP2	0.112					DWI_a24	0.108	DWI_9a	0.072				
SA_11l	0.100	SA_EC	0.056					DWI_d32	0.110	DWI_a10p	0.066				
SA_13l	0.101	SA_PeEc	0.047					DWI_8BM	0.103	DWI_11l	0.072				
SA_OFC	0.091	SA_STSdp	0.059					DWI_p32	0.120	DWI_13l	0.072				
SA_47s	0.061	SA_STSvp	0.058					DWI_10r	0.135	DWI_47s	0.098				
SA_6a	0.072	SA_TE1p	0.113					DWI_47m	0.136	DWI_LIPd	0.074				
SA_i68	0.064	SA_TF	0.045					DWI_8Av	0.134	DWI_6a	0.094				
SA_s68	0.041	SA_TE2p	0.084					DWI_8Ad	0.139	DWI_i68	0.096				
SA_OP1	0.070	SA_PHT	0.090					DWI_9m	0.113	DWI_s68	0.116				
SA_OP23	0.056	SA_PH	0.084					DWI_8BL	0.114	DWI_43	0.082				
SA_52	0.081	SA_PGp	0.047					DWI_9p	0.125	DWI_OP4	0.087				
SA_TA2	0.076	SA_IP1	0.084					DWI_10d	0.091	DWI_OP1	0.096				
SA_FOP4	0.057	SA_PFm	0.042					DWI_8C	0.116	DWI_OP23	0.073				
SA_Pir	0.052	SA_VMV1	0.072					DWI_44	0.136	DWI_52	0.058				
SA_FOP3	0.064	SA_PHA2	0.051					DWI_45	0.111	DWI_RI	0.066				
SA_FOP2	0.051	SA_FST	0.049					DWI_47l	0.115	DWI_PFcm	0.072				
SA_PFt	0.110	SA_25	0.056					DWI_a47r	0.116	DWI_PoI2	0.057				
SA_AIP	0.071	SA_s32	0.053					DWI_6r	0.124	DWI_TA2	0.054				
SA_PreS	0.053	SA_pOFC	0.060					DWI_IFJa	0.111	DWI_FOP4	0.069				
SA_H	0.046	SA_PoI1	0.052					DWI_IFJp	0.115	DWI_MI	0.071				
SA_ProS	0.078	SA_Ig	0.080					DWI_IFSp	0.103	DWI_Pir	0.054				
SA_PeEc	0.047	SA_p10p	0.067					DWI_IFSa	0.082	DWI_AVI	0.061				
SA_STGa	0.042	SA_p47r	0.085					DWI_p946v	0.111	DWI_AAIC	0.049				
SA_PHA1	0.069	SA_MBelt	0.072					DWI_46	0.135	DWI_FOP1	0.069				
SA_STSvp	0.060	SA_TE1m	0.073					DWI_a946v	0.129	DWI_FOP3	0.069				
SA_TE1p	0.096	SA_PI	0.079					DWI_946d	0.129	DWI_FOP2	0.067				
SA_TF	0.045	SA_a32pr	0.045					DWI_9a	0.114	DWI_PFt	0.066				

SA_TE2p	0.071	DWI_10v	0.127	DWI_AIP	0.078
SA_PHT	0.131	DWI_a10p	0.124	DWI_EC	0.059
SA_PH	0.099	DWI_10pp	0.114	DWI_H	0.057
SA_TPOJ1	0.074	DWI_11l	0.152	DWI_PeEc	0.055
SA_TPOJ2	0.080	DWI_13l	0.148	DWI_STGa	0.063
SA_DVT	0.048	DWI_OFC	0.124	DWI_PBelt	0.071
SA_IP2	0.069	DWI_47s	0.139	DWI_A5	0.076
SA_IP1	0.070	DWI_LIPd	0.126	DWI_PHA3	0.074
SA_PFop	0.051	DWI_6a	0.140	DWI_STSda	0.075
SA_PFm	0.058	DWI_i68	0.135	DWI_STSdp	0.086
SA_VMV1	0.110	DWI_s68	0.133	DWI_STSvp	0.088
SA_PHA2	0.049	DWI_43	0.132	DWI_TGd	0.057
SA_V4t	0.089	DWI_OP4	0.148	DWI_TE1a	0.066
SA_FST	0.116	DWI_OP1	0.156	DWI_TE1p	0.104
SA_V3CD	0.093	DWI_OP23	0.133	DWI_TE2a	0.089
SA_LO3	0.099	DWI_52	0.114	DWI_TF	0.091
SA_VMV2	0.071	DWI_RI	0.109	DWI_TE2p	0.098
SA_25	0.048	DWI_PFcm	0.117	DWI_PHT	0.100
SA_s32	0.047	DWI_PoI2	0.118	DWI_PH	0.094
SA_pOFC	0.066	DWI_TA2	0.111	DWI_TPOJ1	0.079
SA_PoI1	0.070	DWI_FOP4	0.135	DWI_TPOJ2	0.086
SA_MBelt	0.096	DWI_MI	0.131	DWI_TPOJ3	0.055
SA_TE1m	0.088	DWI_Pir	0.092	DWI_PGp	0.052
SA_PI	0.077	DWI_AVI	0.126	DWI_IP2	0.073
SA_a32pr	0.059	DWI_AAIC	0.075	DWI_IP1	0.073
		DWI_FOP1	0.128	DWI_IP0	0.054
		DWI_FOP3	0.132	DWI_PFop	0.067
		DWI_FOP2	0.122	DWI_PF	0.059
		DWI_PFt	0.123	DWI_PFm	0.075
		DWI_AIP	0.135	DWI_PGi	0.077
		DWI_EC	0.120	DWI_PGs	0.081
		DWI_PreS	0.126	DWI_V6A	0.050
		DWI_H	0.127	DWI_PHA2	0.057
		DWI_ProS	0.126	DWI_V4t	0.062
		DWI_PeEc	0.126	DWI_FST	0.079
		DWI_STGa	0.093	DWI_31pd	0.051
		DWI_PBelt	0.123	DWI_31a	0.052
		DWI_A5	0.127	DWI_VVC	0.064
		DWI_PHA1	0.122	DWI_s32	0.055
		DWI_PHA3	0.137	DWI_PoI1	0.070
		DWI_STSda	0.122	DWI_Ig	0.077
		DWI_STSdp	0.132	DWI_FOP5	0.056
		DWI_STSvp	0.127	DWI_p10p	0.078
		DWI_TGd	0.096	DWI_p47r	0.073
		DWI_TE1a	0.109	DWI_TGv	0.062
		DWI_TE1p	0.139	DWI_MBelt	0.069
		DWI_TE2a	0.122	DWI_LBelt	0.064
		DWI_TF	0.126	DWI_A4	0.068
		DWI_TE2p	0.130	DWI_STSva	0.077
		DWI_PHT	0.164	DWI_TE1m	0.082
		DWI_PH	0.143	DWI_PI	0.063
		DWI_TPOJ1	0.128	DWI_cauda	0.058
		DWI_TPOJ2	0.149	DWI_hippo	0.053
		DWI_TPOJ3	0.129	DWI_palli	0.063
		DWI_DVT	0.122	DWI_puta	0.066
		DWI_PGp	0.125	DWI_thala	0.059
		DWI_IP2	0.130	DWI_ventdc	0.060
		DWI_IP1	0.134		
		DWI_IP0	0.122		
		DWI_PFop	0.109		
		DWI_PF	0.116		
		DWI_PFm	0.141		
		DWI_PGi	0.141		
		DWI_PGs	0.142		
		DWI_V6A	0.114		
		DWI_VMV1	0.125		
		DWI_VMV3	0.118		
		DWI_PHA2	0.146		
		DWI_V4t	0.128		
		DWI_FST	0.150		
		DWI_V3CD	0.107		
		DWI_LO3	0.132		
		DWI_VMV2	0.114		
		DWI_31pd	0.118		
		DWI_31a	0.130		
		DWI_VVC	0.120		
		DWI_25	0.101		
		DWI_s32	0.122		
		DWI_pOFC	0.098		
		DWI_PoI1	0.125		
		DWI_Ig	0.124		
		DWI_FOP5	0.118		
		DWI_p10p	0.126		
		DWI_p47r	0.118		
		DWI_TGv	0.102		
		DWI_MBelt	0.135		
		DWI_LBelt	0.123		
		DWI_A4	0.128		
		DWI_STSva	0.112		
		DWI_TE1m	0.132		
		DWI_PI	0.116		
		DWI_a32pr	0.119		
		DWI_p24	0.101		
		DWI_accumb	0.103		
		DWI_amy	0.107		
		DWI_cauda	0.116		
		DWI_hippo	0.122		
		DWI_palli	0.132		
		DWI_puta	0.118		
		DWI_thala	0.118		
		DWI_ventdc	0.122		

Supplementary Table 3 - **Path b coefficients**. Coefficients from the b path (brain - IST 2000 R score) of the mediation analysis after re-estimation with lavaan. Analyses were performed twice, controlling for one of the PGS (EA, GI). Results from the SA analysis are depicted in green, results from the CT analysis in blue and results from the DWI analysis in red. There were no brain areas whose rsfMRI showed any association with I-ST 200 R score.

PGS-EA		PGS-GI		PGS-EA		PGS-GI		PGS-EA		PGS-GI	
**UV**	**b**	**UV**	**b**	**UV**	**b**	**UV**	**b**	**UV**	**b**	**UV**	**b**
SA_4	0,078	SA_MIP	0,056	CT_FOP1	0,122			DWI_LO1	-0,050	DWI_45	0,338
SA_5mv	-0,120	SA_2	-0,190	CT_PreS	0,120			DWI_LO2	-0,102	DWI_Pir	-0,138
SA_5L	0,009	SA_45	0,109	CT_H	0,086			DWI_5mv	-0,251	DWI_VMV1	-0,110
SA_7PC	-0,080	SA_OFC	0,089	CT_TE2a	-0,104			DWI_6ma	0,153		
SA_MIP	0,065	SA_OP1	0,060	CT_25	-0,048			DWI_44	0,068		
SA_1	-0,227	SA_STGa	0,068	CT_pOFC	-0,113			DWI_45	0,164		
SA_2	0,037	SA_PH	0,060	CT_PI	0,100			DWI_6r	0,136		
SA_45	0,049	SA_IP1	0,051					DWI_47s	0,234		
SA_6r	0,055							DWI_43	0,159		
SA_IFSa	0,121							DWI_Pir	-0,210		
SA_OFC	0,064							DWI_VMV1	-0,157		
SA_OP1	0,059							DWI_pOFC	-0,057		
SA_FOP3	0,035										
SA_EC	0,094										
SA_ProS	-0,091										
SA_STGa	0,078										
SA_PH	0,068										
SA_IP1	0,043										

Supplementary Table 4 **- Mediation coefficients**. Coefficients of the mediation analysis after re-estimation with lavaan. Tables show coefficients for a, b and ab. Analyses were performed two times, with three different PGS as IV (EA, GI). Results from the SA analysis are depicted in green, results from the CT analysis in blue and results from the DWI analysis in red. There are no areas whose rsfMRI connectivity mediated the effect of any of the PGS on I-S-T 2000 R score.

PGS-EA				PGS-GI				PGS-EA				PGS-GI				PGS-EA				PGS-GI			
**UV**	**a**	**b**	**ab**	**UV**	**a**	**b**	**ab**	**UV**	**a**	**b**	**ab**	**UV**	**a**	**b**	**ab**	**UV**	**a**	**b**	**ab**	**UV**	**a**	**b**	**ab**
SA_4	0.094	0.063	0.006	SA_MIP	0.083	0.108	0.009	CT_FOP1	0.115	0.122	0.014					DWI_LO1	0.136	-0.050	-0.007	DWI_45	0.054	0.305	0.017
SA_MIP	0.076	0.040	0.003	SA_PH	0.084	0.110	0.009	CT_PreS	0.069	0.105	0.007					DWI_LO2	0.108	-0.102	-0.011	DWI_Pir	0.054	-0.203	-0.011
SA_1	0.059	-0.226	-0.013	SA_IP1	0.084	0.035	0.003	CT_25	0.075	-0.123	-0.009					DWI_5mv	0.125	-0.251	-0.031				
SA_6r	0.067	0.047	0.003													DWI_6ma	0.140	0.153	0.021				
SA_IFSa	-0.073	0.119	-0.009													DWI_44	0.136	0.068	0.009				
SA_OFC	0.091	0.060	0.005													DWI_45	0.111	0.164	0.018				
SA_OP1	0.070	0.055	0.004													DWI_6r	0.124	0.136	0.017				
SA_STGa	0.042	0.073	0.003													DWI_47s	0.139	0.234	0.033				
SA_PH	0.099	0.058	0.006													DWI_43	0.132	0.159	0.021				
SA_IP1	0.070	0.066	0.005													DWI_Pir	0.092	-0.210	-0.019				
																DWI_VMV1	0.125	-0.157	-0.020				
																DWI_pOFC	0.098	-0.057	-0.006				

Supplementary Table 5 **- Mediation coefficients for the hemispheric-specific analysis**. See supplenentary figures 4 and 5. Coefficients of the mediation analysis after re-estimation with lavaan. Tables show coefficients for a, b and ab. Analyses were performed two times, with three different PGS as IV (EA, GI). Results from the SA analysis are depicted in green, results from the CT analysis in blue and results from the DWI analysis in red. There are no areas whose rsfMRI connectivity mediated the effect of any of the PGS on I-S-T 2000 R score.

PGS-EA				PGS-GI				PGS-EA				PGS-GI				PGS-EA				PGS-GI			
**UV**	**a**	**b**	**ab**	**UV**	**a**	**b**	**ab**	**UV**	**a**	**b**	**ab**	**UV**	**a**	**b**	**ab**	**UV**	**a**	**b**	**ab**	**UV**	**a**	**b**	**ab**
SURF_L_4	0.084	0.080	0.007					THICK_L_FOP1	0.101	0.118	0.012					DWI_L_LO1	0.138	-0.104	-0.014	DWI_L_45	0.051	0.096	0.005
SURF_L_IFSa	-0.064	0.106	-0.007					THICK_L_25	0.047	-0.127	-0.01					DWI_L_45	0.106	0.184	0.020	DWI_R_45	0.051	0.065	0.003
SURF_L_PoI2	0.044	0.076	0.003					THICK_R_PreS	0.098	0.115	0.011					DWI_L_43	0.118	0.187	0.022				
SURF_R_MIP	0.065	0.085	0.006					THICK_R_H	0.071	0.113	0.008					DWI_L_25	0.099	-0.145	-0.014				
								THICK_R_IP2	-0.05	-0.12	0.006					DWI_R_V3B	0.095	-0.154	-0.015				
																DWI_R_45	0.102	0.130	0.013				
